# Neuronal TIMP2 regulates hippocampus-dependent plasticity and extracellular matrix complexity

**DOI:** 10.1038/s41380-023-02296-5

**Published:** 2023-11-02

**Authors:** Ana Catarina Ferreira, Brittany M. Hemmer, Sarah M. Philippi, Alejandro B. Grau-Perales, Jacob L. Rosenstadt, Hanxiao Liu, Jeffrey D. Zhu, Tatyana Kareva, Tim Ahfeldt, Merina Varghese, Patrick R. Hof, Joseph M. Castellano

**Affiliations:** 1 Nash Family Department of Neuroscience, Friedman Brain Institute, Icahn School of Medicine at Mount Sinai, New York, NY USA; 2 Ronald M. Loeb Center for Alzheimer’s Disease, Icahn School of Medicine at Mount Sinai, New York, NY USA; 3 Department of Neurology, Icahn School of Medicine at Mount Sinai, New York, NY USA; 4 Black Family Stem Cell Institute, Icahn School of Medicine at Mount Sinai, New York, NY USA; 5 Graduate School of Biomedical Sciences, Icahn School of Medicine at Mount Sinai, New York, NY USA

**Keywords:** Neuroscience, Molecular biology

## Abstract

Functional output of the hippocampus, a brain region subserving memory function, depends on highly orchestrated cellular and molecular processes that regulate synaptic plasticity throughout life. The structural requirements of such plasticity and molecular events involved in this regulation are poorly understood. Specific molecules, including tissue inhibitor of metalloproteinases-2 (TIMP2) have been implicated in plasticity processes in the hippocampus, a role that decreases with brain aging as expression is lost. Here, we report that TIMP2 is highly expressed by neurons within the hippocampus and its loss drives changes in cellular programs related to adult neurogenesis and dendritic spine turnover with corresponding impairments in hippocampus-dependent memory. Consistent with the accumulation of extracellular matrix (ECM) in the hippocampus we observe with aging, we find that TIMP2 acts to reduce accumulation of ECM around synapses in the hippocampus. Moreover, its deletion results in hindrance of newborn neuron migration through a denser ECM network. A novel conditional TIMP2 knockout (KO) model reveals that neuronal TIMP2 regulates adult neurogenesis, accumulation of ECM, and ultimately hippocampus-dependent memory. Our results define a mechanism whereby hippocampus-dependent function is regulated by TIMP2 and its interactions with the ECM to regulate diverse processes associated with synaptic plasticity.

## Introduction

Cellular, molecular, and functional changes have been documented in the brain as aging progresses, leaving it susceptible to diseases like Alzheimer’s disease. Since aging serves as the major risk factor for many neurological disorders, aging processes have increasingly been considered to be valid therapeutic targets. Growing evidence in animal models indicates that the aged brain can be rejuvenated through manipulation of the systemic environment, including through young plasma transfer or sharing of young blood through heterochronic parabiosis [1–4]. Aged mice exposed to young blood exhibit improved synaptic plasticity and cognitive performance [2]. We and others have begun to identify factors present in young blood that confer brain rejuvenation phenotypes in aged mice [3, 4]. Among those, we showed that tissue inhibitor of metalloproteinases-2 (TIMP2), a protein elevated early in life, revitalizes function of the aged mouse hippocampus [4]. Despite demonstrating that this factor is critical for young plasma-mediated memory improvements in aged mice [4], little is known of the cellular and molecular details connecting TIMP2 to hippocampal function.

TIMP2 is a secreted protein ~21 kDa in size, primarily known for its role in regulating peripheral extracellular matrix (ECM) remodeling [5] through its unique dual roles in regulating activation of latent matrix metalloproteinases (MMPs), including MMP2, to proteolytically active forms, and the inhibition of MMP activity [6, 7]. Through these roles, TIMP2 has been shown to regulate maintenance of ECM network structure in many tissues with myriad disease implications, including heart failure [8], cancer [9], and ischemic stroke [10], among others. Early reports described expression of TIMP2 from development into adulthood [11] and its role in regulating cortical neuronal differentiation [12] and subtle motor phenotypes [13], but few studies have examined the biological role of TIMP2 in the adult brain and how it modulates function. While we recently demonstrated that TIMP2 revitalizes overall hippocampus-dependent cognitive function in aged mice [4], the biological role of TIMP2 expressed within the hippocampus has not been studied. TIMP2 belongs to a small family of tissue metalloproteinase inhibitors that includes TIMP1, TIMP3, and TIMP4, all of which perform broad MMP targeting functions but in diverse contexts in the brain, including in myelin repair [14], neurovascular regulation [15], or in regulation of the tumor microenvironment [16]. Interestingly, TIMP2 shares only ~40–50% sequence homology with other TIMP family members and is particularly expressed in the brain and enriched specifically in  the hippocampus relative to other TIMPs. Since levels of TIMP2 decline in the hippocampus with age [4], understanding the function of this local source will be important to understand its normal function and position it for putative therapies for age-related brain disorders. We further sought to understand the extent to which ECM regulation is related to disparate processes of plasticity to regulate complex behavioral outputs.

Here we define a mechanism by which TIMP2 regulates ECM components in the hippocampus in a manner that regulates processes linked to plasticity and memory. We find that TIMP2 is predominantly expressed by neurons in the hippocampus and is present at high levels in the brain extracellular space. Deletion of TIMP2 induces transcriptomic changes in the hippocampus that are consistent with processes related to synaptic plasticity and adult neurogenesis. We find that loss of TIMP2 results in fewer dendritic spines in dentate gyrus (DG) granule cells, decreased adult DG neurogenesis along several stages, and impaired memory in various hippocampus-dependent tasks. Loss of TIMP2 also causes an accumulation of ECM proteins, particularly around synapses within the DG, and impaired migration of neuroblasts within the neurogenic niche, reflecting dysregulated ECM turnover and mimicking ECM accumulation seen in the aged brain. Finally, we developed a mouse model for conditional deletion of TIMP2 from neurons that exhibits the functional phenotypes observed in TIMP2 KO mice, further arguing for a role of the neuronal pool of TIMP2 in shaping plasticity-related function of the hippocampus. Our findings highlight a mechanism by which neuronal TIMP2 remodeling of the ECM promotes synapse plasticity and is required for memory.

## Materials and methods

See *Supplementary Information* for additional details.

### Animals

All animal procedures were performed in accordance with the National Institutes of Health Guide for Care and Use of Laboratory Animals and the Icahn School of Medicine at Mount Sinai Institutional Animal Care and Use Committee. TIMP2 knockout (KO) (Jackson Laboratory) and WT female mice or Syn^Cre/+^; TIMP2^fl/fl^ and TIMP2^fl/fl^ mice (generated as described in Supplementary Information) were used at 2–3 months or 5–6 months of age unless indicated otherwise. Littermate controls were used for all experiments. Mice were maintained on a 12 h light/dark cycle at constant temperature (23 °C) with ad libitum access to food and water.

### Bulk RNA-sequencing

RNA-sequencing was performed on hippocampi dissected from WT and TIMP2 KO male and female mice. Quality of extracted RNA (RNeasy Mini Kit, Qiagen) was measured by Agilent TapeStation Bioanalyzer (Agilent Technologies), and all samples exhibited RNA Integrity Number (RIN) > 8. cDNA libraries were prepared with poly(A) selection and sequenced using Illumina Hiseq (2 × 150 bp paired-end) (Genewiz). At least 25 M clean reads were generated from each sample and mapped to the *Mus musculus* GRCm38 reference genome available on ENSEMBL, using STAR aligner (v.2.5.2b). After extraction of gene hit counts, DESeq2 was used for downstream differential expression analysis. Differentially expressed genes (DEGs; nominal *P* < 0.05*)* were used for Gene Set Enrichment Analysis (GSEA; https://www.gsea-msigdb.org/gsea/index.jsp; last accessed Spring 2023). Volcano plot was generated with R (version 4.1.2). Weighted gene co-expression network analysis (WGCNA) [17] was performed on all genes to identify modules with coordinated expression patterns according to TIMP2 genotype. Significant modules (*P* < 0.05) were analyzed by GSEA to examine gene ontology enrichment. RNA-seq data files are available as GEO accession GSE223188.

### Animal procedures

Mice were injected intraperitoneally (i.p.) once with 150 mg/kg of 5-bromo-2′-deoxyuridine (BrdU; Sigma) 24 h before sacrifice for cell proliferation assessment, or daily for 5 days, followed by sacrifice/perfusion 28 days after initial injection, for cell fate analysis. In vivo microdialysis proceeded according to previous methods [18] with some adaptations (described in Supplementary Information).

### Immunohistochemistry

For all immunohistochemistry experiments, mice were anesthetized with a cocktail of ketamine (90 mg/kg) and xylazine (10 mg/kg) and transcardially perfused with ice-cold 0.9% saline, and brains were postfixed in 4% paraformaldehyde and preserved with 30% sucrose before sectioning at 40 µm on a freezing-sliding microtome (SM2010R, Leica). Sections were incubated overnight with indicated primary antibodies, followed by fluorescent secondary antibodies. Image processing was performed with LSM 780 confocal microscope (Zeiss) using 40×/1.4 Oil DIC objective. Four equally-spaced sections per mouse were used to count the total number of positive cells within DG (subgranular zone and hilar subregions) using stereological principles. All counts were performed using FIJI in a blinded fashion, according to similar methods [4].

### Aggrecan and Homer1 puncta quantification

Quantification of the number of puncta by super-resolution microscopy proceeded according to similar methods [19]. Images were acquired by confocal imaging using LSM 880 with AiryScan in super-resolution mode (Zeiss) set with a 63X/1.4 Oil DIC objective with 5X optical zoom. Aggrecan puncta were quantified with Puncta Analyzer plugin [20] in ImageJ, and thresholding was applied equally across images. Colocalization of co-stained Aggrecan and Homer1 puncta was analyzed with the Puncta Analyzer plugin with a minimum pixel specification of 4. Three images in the molecular layer of the DG were averaged per mouse for analysis.

### Dendritic spine analysis by iontophoretic dye injections

Tissue was processed according to similar work [21], and then coronal sections were incubated in 250 ng/ml DAPI to enable DG identification. Sections were mounted on nitrocellulose membrane filters, immersed in ice-cold PB, and DG granule cells were iontophoretically injected with 5% Lucifer Yellow (Invitrogen) under a direct current of 3–8 nA until the dye filled distal ends of the dendrites, and then sections were mounted between spacers placed on gelatin-coated glass slides (#22-214-320, Thermo Fisher). Images of dendritic segments from DG granule cells at the suprapyramidal blade were acquired on a Zeiss LSM 780 confocal microscope using a 100x/1.46 Oil DIC M27 Plan-Apochromat objective, and stacks were acquired at 512 × 512-pixel resolution with a Z-step of 0.1 µm, optical zoom of 3.3×, pinhole setting of 1 Airy Unit, and optimal settings for gain and offset. Three z-stacks were imaged from each neuron. Confocal stacks were deconvolved using an iterative blind deconvolution algorithm (AutoQuant X, vX3.0.1, MediaCybernetics). Deconvolved stacks were analyzed using Neurolucida 360 (v2019.2.1; MBF Bioscience) for semi-automated reconstruction to determine spine density and morphology. Spines were classified as stubby, thin, mushroom, and filopodia, according to previous work [21, 22]. 4–5 mice per genotype, 6 neurons per mouse, and 3 dendrites per neuron were analyzed.

### Hippocampus-dependent behavior

#### Novel location recognition

Mice were habituated to the open-field arena for 6 min, following exposure to two different objects in fixed positions for three consecutive trials of 6 min each. On day 2, mice explored the same arena with one object displaced to a novel position. Time spent exploring each object was manually scored in a blinded fashion to assess the discrimination index for the novel location.

#### Contextual fear-conditioning

Mice were trained using a 2-shock contextual fear conditioning paradigm, as previously described [4]. Mice received two periods of 30 s consisting of a paired cue light and a tone of 1000 Hz, followed by a light foot-shock (2 s, 0.5 mA) separated by a 180 s interval (light foot-shock; Ugo Basile). Twenty-four hours later, mice were re-exposed to the same context for 3 min, and freezing levels (contextual) were measured using EthoVision XT system software (v14.0.1319, Noldus).

#### Barnes maze

Mice were tested on a circular Barnes maze in which mice navigate using visual cues to an alternating escape hole over four trials on each day of the task, as described [4]. Search strategy classification was manually performed based on methods adapted from previous work [23], categorized as localized, serial, random, scanning, focal, focal missense, targeted, and direct. Each related strategy was categorized into non-hippocampus-dependent strategies (localized, serial, random, and scanning), and hippocampus-dependent strategies (focal, focal missense, targeted, and direct). Cognitive performance of each trial on day 3 was scored according to the following, with a separation of 1 point between non-hippocampus-dependent and hippocampus-dependent strategies to account for additional cognitive complexity: localized=0, serial=1, random=2, scanning=3, focal=5, focal missense=5, targeted=6, direct=6. Focal and focal missense strategies are assigned “5”, as both are goal-directed strategies used without achieving the final target. Targeted and direct strategies were assigned “6” since both are goal-directed strategies with successful achievement of the final target.

### Statistical analysis

Aside from RNA-seq analysis, statistical analyses were performed using GraphPad Prism version 9.0 software (GraphPad Software) using tests described in figure legends. All experiments were performed in a blinded fashion, and randomization was performed where applicable. Some schematic illustrations were adapted from Biorender tools.

## Results

### TIMP2 is expressed in hippocampal neurons, and its deletion induces transcriptomic changes

**Fig. 1:**
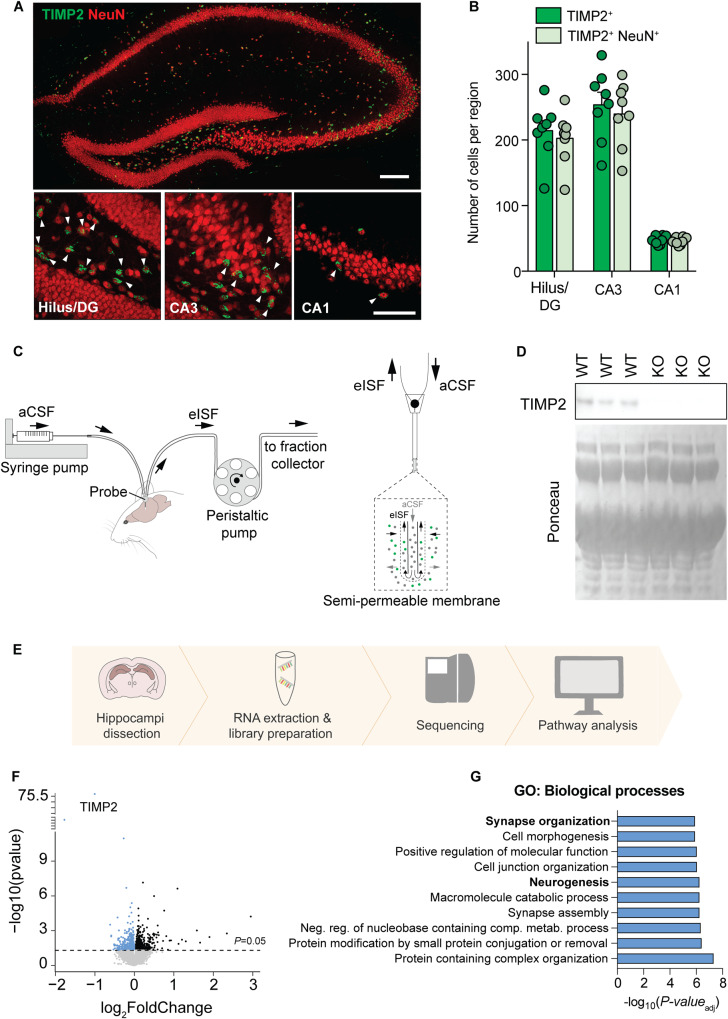
TIMP2 is expressed in neurons of the hippocampus, and its deletion induces transcriptomic changes. **A** Low-magnification view (upper image) of mouse hippocampus and high-magnification view (lower images) of hilus/DG, CA3, and CA1 subregions showing TIMP2^+^ cells and TIMP2^+^ cells staining for pan-neuronal nuclei marker NeuN. Scale bars, 200 μm and 20 μm (inset). **B** Quantification of the total number of TIMP2^+^ cells and TIMP2^+^ NeuN^+^ cells across hippocampal subregions in WT mice (2 months of age; *N* = 8, males and females). **C** Schematic representation of high molecular-weight cut-off (1-MDa) in vivo microdialysis for assessing TIMP2 levels in mouse hippocampal ISF. **D** TIMP2 immunoblotting of hippocampal ISF dialyzed from 2-month-old WT and TIMP2 KO mice, with corresponding Ponceau S stain. **E** Schematic representation of bulk RNA-seq workflow performed in isolated WT and TIMP2 KO hippocampi (*N* = 13–17 mice/group, sex-matched) for transcriptomic analysis. **F** Volcano plot showing the fold-change of genes (log_2_ scale) differentially expressed in hippocampus of TIMP2 KO vs. WT mice. Downregulated DEGs at nominal *P* < 0.05 are highlighted in blue (upregulated in black). **G** Top 10 significant pathways for downregulated DEGs from Gene Set Enrichment Analysis. Data are represented as mean ± SEM. DG dentate gyrus, eISF exchangeable interstitial fluid, aCSF artificial cerebrospinal fluid.

TIMP2 exhibits high expression in the hippocampus relative to other brain regions [4]. To identify the cellular sources of TIMP2 in the hippocampus and to begin to probe its function in this region, we examined TIMP2 protein levels by confocal microscopy in various subfields of the hippocampus of 2-month-old wildtype (WT) male and female mice. We found that TIMP2 is expressed in the hilus of dentate gyrus (DG), as well as CA3 and CA1 subfields of the hippocampus, and we found that the majority of TIMP2-expressing cells also stain for pan-neuronal marker, NeuN, arguing that the major cellular source of TIMP2 expression in these areas was neuronal (Fig. 1A, B). We did not detect differential TIMP2 neuronal expression in the hilus/DG, CA3, and CA1 subfields between males and females (Supplementary Fig. 1A–C).

Given the high level of TIMP2 expression seen in neurons in the hippocampus, we characterized levels in the extracellular space adjacent to this cellular source. To measure extracellular levels of TIMP2 in this region, we implanted high molecular-weight cut-off (1 MDa) probes into hippocampi of freely moving, 2–3-month-old WT mice to measure interstitial fluid (ISF) levels of TIMP2 by in vivo microdialysis. TIMP2 was readily detectable in the ISF of hippocampus (Fig. 1D), and extrapolated values of exchangeable ISF TIMP2 were ~10.1 ± 0.16 ng/mL (*N* = 3) at a flow rate of 1 μl/min. Importantly, the 21–22 kDa TIMP2 band was absent in the ISF of hippocampus from mice in which TIMP2 has been deleted (KO) (Fig. 1D).

The high levels of TIMP2 within neurons and the extracellular space of the hippocampus argues for unappreciated roles for its function locally in this region, prompting us to examine the cellular and molecular processes regulated by TIMP2. To identify putative pathways involved in TIMP2 function in the hippocampus, we isolated hippocampi from WT and TIMP2 KO male and female mice and processed the tissue for RNA-sequencing (Fig. 1E). A total of 905 differentially expressed genes (DEGs; nominal *P* < *0.05*) were identified in TIMP2 KO compared to WT (Fig. 1F), with TIMP2 being the top DEG, as expected. Of these genes, 449 DEGs were downregulated in TIMP2 KO hippocampi (Fig. 1F). To gain insights into how the altered transcriptome may reflect altered biological processes, we performed gene set enrichment analysis (GSEA) [24]. Downregulated DEGs in TIMP2 KO were enriched for a number of Gene Ontology (GO) Biological Processes related to plasticity, including “cell morphogenesis”, “neurogenesis”, and “synapse organization” (Fig. 1G). Upregulated DEGs in TIMP2 KO were enriched in functions that included “regulation of cell death”, among others (Supplementary Fig. 1E). Using an independent and unsupervised approach, weighted gene co-expression network analysis (WGCNA), to understand pathways disrupted in TIMP2 KO hippocampus, we found several significant modules of correlated genes (Supplementary Fig. 1F). The gene set from the most significant downregulated module, “pale turquoise”, was significantly enriched for Biological Processes and Cellular Components related to “neurogenesis”, “dendritic tree”, and “synapse” (Supplementary Fig. 1G, H), reinforcing that TIMP2 may be involved in plasticity processes.

### TIMP2 is necessary for adult neurogenesis in the dentate gyrus

**Fig. 2:**
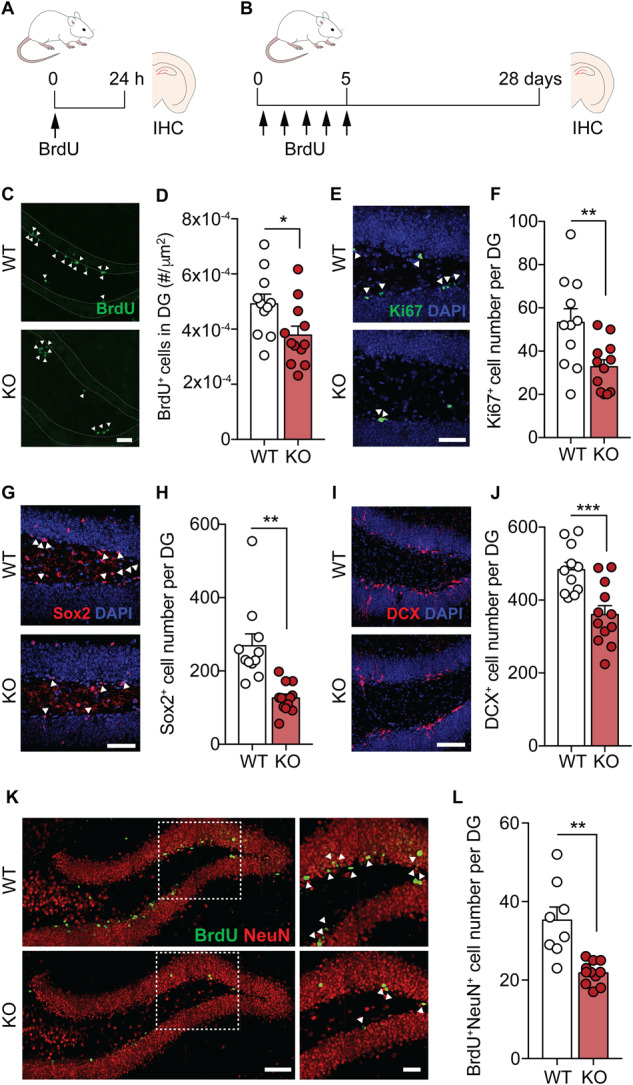
TIMP2 is necessary for adult neurogenesis in the normal dentate gyrus. **A** Schematic timeline of BrdU intraperitoneal injection protocol to label proliferating cells in the DG in isolated brain sections. **B** Schematic timeline of BrdU intraperitoneal injection protocol used for cell fate “survival” labeling in the DG of isolated brain sections. **C** Representative confocal microscopy images of BrdU^+^ cells in the DG of WT and TIMP2 KO mice (2–3 months of age, *N* = 11–12 mice per group; arrowheads indicate BrdU^+^ cells; scale bar, 50 μm) with corresponding (**D**) quantification of number of BrdU^+^ cells in the DG per unit area. **E** Representative confocal microscopy images of proliferating Ki67^+^ cells in the DG of WT and TIMP2 KO mice (2–3 months of age, *N* = 11–12 mice per group; arrowheads indicate Ki67^+^ cells; scale bar, 50 μm) with corresponding (**F**) quantification of the number of Ki67^+^ proliferating cells per DG in WT and TIMP2 KO mice. **G** Representative confocal microscopy images of Sox2^+^ cells in the DG of WT and TIMP2 KO mice (2–3 months of age, *N* = 11–12 mice per group; arrowheads indicate Sox2^+^ cells; scale bar, 50 μm) with corresponding (**H**) quantification of Sox2^+^ neural progenitor cells in the DG of WT and TIMP2 KO mice. **I** Representative confocal microscopy images of DCX^+^ cells in the DG of WT and TIMP2 KO mice (2–3 months of age, *N* = 11–12 mice per group; scale bar, 50 μm) with corresponding (**J**) quantification of DCX^+^ immature neuroblasts in DG of WT and TIMP2 KO mice. **K** Representative confocal microscopy images of BrdU^+^ NeuN^+^ cells in DG of WT and TIMP2 KO mice (2–3 months of age. *N* = 8–11 mice per group; scale bar, 100 μm and 50 μm (inset)) with corresponding (**L**) quantification of newborn neurons (BrdU^+^ NeuN^+^ cells) in the DG of WT and TIMP2 KO mice. Data are represented as mean ± SEM. Student’s *t* test for two-group comparisons (with Welch’s correction for (**H**) and (**L**)). *P < 0.05; **P < 0.01; ***P < 0.001. Data points represent individual mice. IHC immunohistochemistry, DG dentate gyrus, DCX doublecortin.

To evaluate the extent to which our discovery-based pathway approach from RNA-seq highlighted processes that may be regulated by TIMP2, we examined the key cellular processes implicated. As one of the top downregulated pathways in hippocampus of TIMP2 KO vs. WT mice was “neurogenesis”, we used confocal microscopy to begin to address whether adult neurogenesis processes within the DG are indeed altered by absence of TIMP2. To examine whether TIMP2 regulates overall cell proliferation, we injected WT and TIMP2 KO mice with the proliferation marker bromodeoxyuridine (BrdU), which labels the pool of proliferating cells in the subgranular zone (SGZ) of DG (Fig. 2A). We found that the DG of TIMP2 KO mice had significantly fewer proliferating cells than in WT mice (Fig. 2C), as quantified by the total number of BrdU^+^ cells (Fig. 2D). We observed a similar result when analyzing the pool of Ki67^+^ cells in the SGZ (Fig. 2E, F), which marks a larger pool of proliferating cells in other phases. We next analyzed the pool of neural progenitor cells and the immature neuroblasts they ultimately give rise to, using SRY-Box Transcriptional Factor 2 (Sox2) and doublecortin (DCX) as markers, respectively. Deletion of TIMP2 resulted in a significant decrease in the number of Sox2^+^ neural progenitor cells (Fig. 2G, H), and of DCX^+^ immature neurons (Fig. 2I, J). In the subventricular zone (SVZ), a neurogenic niche in which we found TIMP2 levels are low (Supplementary Fig. 2A), proliferation assessed by BrdU^+^ cell number was indistinguishable between TIMP2 KO and WT mice (Supplementary Fig. 2B, C).

To examine whether the disrupted pool of neuroblasts in the DG corresponds to a differing number of surviving immature neurons, we injected TIMP2 KO and WT mice by pulse-chase labeling with BrdU for 5 days and quantified the number of label-retaining, surviving BrdU^+^ NeuN^+^ neurons in the SGZ of DG 4 weeks later (Fig. 2B). Using this paradigm, we found that the DG of TIMP2 KO mice had significantly fewer newborn neurons compared with the DG of WT mice (Fig. 2K, L). Together, these results are consistent with TIMP2-mediated roles in adult neurogenesis and related pathways uncovered by our transcriptomic analysis.

### TIMP2 regulates dendritic spine plasticity in the dentate gyrus

**Fig. 3:**
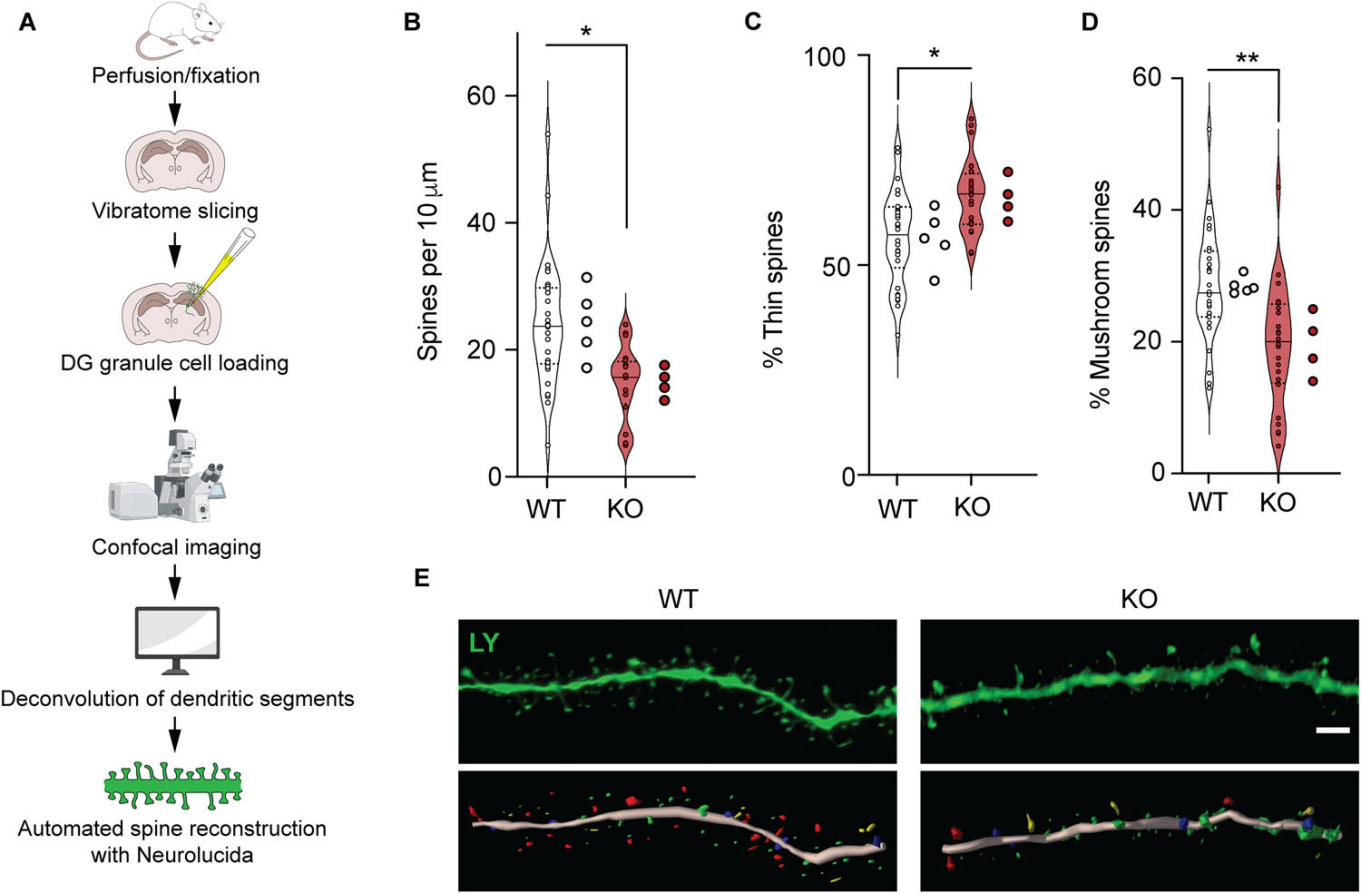
TIMP2 regulates dendritic spine plasticity within the dentate gyrus. **A** Schematic representation of the workflow for dendritic spine quantification in Lucifer Yellow-filled DG granule cells. **B** Overall dendritic spine density in DG granule cells iontophoretically labeled with Lucifer Yellow using sections isolated from six-month-old WT and TIMP2 KO mice (*N* = 6 neurons per mouse from *N* = 4–5 mice per group). **C** Quantification of the percentage of spines categorized according to “thin” spine heads or (**D**) “mushroom” spine heads. **E** Representative deconvolved confocal image of a dendritic segment from WT and TIMP2 KO Lucifer Yellow-labeled brain sections, and the downstream 3D reconstructions, with dendritic segment shown in pink, thin spines in green, stubby in blue, mushroom in red, and filopodia in yellow. Scale bar, 2 μm. Data are represented as mean ± SEM. Nested *t*-test for comparisons with neuron and mouse as levels. *P < 0.05; **P < 0.01. Data points represent neurons (left) and mice (right) for each group. LY Lucifer Yellow.

Our transcriptomic data in TIMP2 KO hippocampi suggest a role for TIMP2 acting within the hippocampus to modulate plasticity processes, including “synapse organization”, possibly at the structural level. To evaluate the impact of TIMP2 deletion on dendritic spines, the cellular substrates of memory storage [25], we turned to a rigorous method to sensitively quantify dendritic spines and classify them. We performed Lucifer Yellow iontophoretic dye-filling of mature DG granule cells in isolated slices from TIMP2 KO and WT mice for high-resolution imaging and reconstruction (Fig. 3A) [22, 26]. We found that neurons in the DG of TIMP2 KO mice exhibited significantly reduced dendritic spine density compared to those in WT DG (Fig. 3B, E). We then used automated classification algorithms [22, 27] to classify and quantify spines based on morphology, as a readout of spine plasticity. Intriguingly, we found a significantly higher proportion of immature thin spines in labeled DG neurons from TIMP2 KO mice compared to those in WT mice (Fig. 3C, E) and a significantly decreased proportion of mature mushroom spines (Fig. 3D, E) exhibited by TIMP2 KO relative to WT DG neurons. Mushroom spines are a particularly stable class of spines with lower turnover [28] and are proposed to serve as structural correlates of memory [29]. Of note, our observations of spines in DG neurons of TIMP2 KO mice are in line with previous descriptions of altered synaptic function upon TIMP2 modulation [4], including long-term potentiation, which was shown to be affected by modulating levels of TIMP2 [4]. Collectively, these results suggest that TIMP2 may act to promote plasticity through regulation of adult neurogenesis and dendritic spine formation.

### TIMP2 is required for hippocampus-dependent memory function

**Fig. 4:**
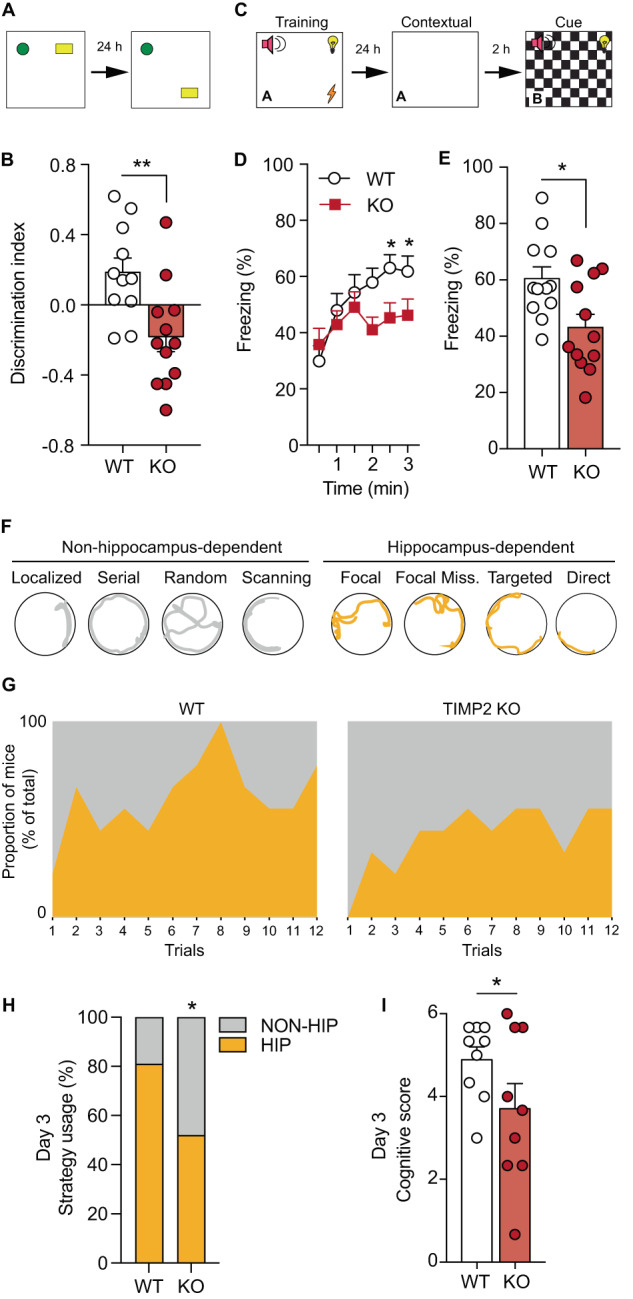
TIMP2 is required for hippocampus-dependent memory function. **A** Schematic of the novel location recognition assay. **B** Discrimination index for novel location recognition on day 2 for WT and TIMP2 KO mice (2–3 months of age, *N* = 11–12 mice per group). **C** Schematic of the contextual and cued fear-conditioning assay. **D**, **E** Freezing levels by interval and overall measured in the conditioned-fear context A in WT and TIMP2 KO mice (2-3 months of age, *N* = 11–12 mice per group). **F** Schematic diagram of modified Barnes maze with color coding for overall strategy classification. **G** Proportion of WT and TIMP2 KO mice using non-hippocampus-dependent (gray) and hippocampus-dependent (yellow) strategies during the testing trials (2–3 months of age, *N* = 9 mice per group). **H** Strategy utilization by WT and TIMP2 KO mice on day 3 of the Barnes Maze and corresponding (**I**) cognitive scores, ranked by strategy complexity for WT and TIMP2 KO mice on day 3 in the Barnes Maze. Data are represented as mean ± SEM. Student’s *t* test for two-group comparisons in (**B**, **D**, **E**), chi-square test in (**H**), and nested *t*-test (**I**) for trial and mouse levels. *P < 0.05, **P < 0.01. Data points represent individual mice.

The hippocampus is critical for spatial memory and learning, likely through regulation of adult neurogenesis in the DG (reviewed in [30]), as well as through changes in synaptic structure [31, 32]. The changes we observed in both adult neurogenesis and dendritic spine plasticity in TIMP2 KO hippocampi prompted us to investigate whether TIMP2 is involved in hippocampus-dependent memory and learning. We first utilized the novel location recognition task (Fig. 4A), in which mice use spatial cues in their arenas to learn and associate the positions of objects in space. Twenty-four hours after training, we observed that TIMP2 KO mice exhibited significantly decreased preference for the training object when displaced, as indicated by the reduced discrimination index (Fig. 4B), thus suggesting a critical role of TIMP2 in spatial memory in healthy mice. We next employed contextual and cued fear-conditioning to evaluate whether the mice differ in the ability to discriminate the previously aversive training context or a new cue-driven context (Fig. 4C). Assessment of context discrimination revealed significantly reduced freezing behavior by TIMP2 KO mice, suggesting impaired hippocampus-associated contextual discrimination in the absence of TIMP2 (Fig. 4D, E), while amygdala-associated cued freezing levels were unchanged (Supplementary Fig. 3A). We next examined whether more complex hippocampus-dependent spatial memory was altered in a Barnes maze assay in which mice use a variety of strategies while navigating an illuminated maze surface to an escape hole using spatial cues. Strategies were divided into non-hippocampus-dependent and hippocampus-dependent with increasing levels of cognitive demand (Fig. 4F) [23]. Analysis of the strategies used to reach the escape hole revealed that TIMP2 KO mice delayed the switch from non-hippocampus-dependent to hippocampus-dependent strategies while performing the task (Fig. 4G). On day 3 of the Barnes maze, a significantly lower proportion of TIMP2 KO mice used hippocampus-dependent strategies compared to WT mice (Fig. 4H). Further categorization of cognitive performance by scoring the different strategies according to level of complexity related to spatial learning [33] showed that TIMP2 KO mice achieved significantly lower cognitive scores relative to WT mice (Fig. 4I), consistent with impaired hippocampus-dependent function in TIMP2 KO mice in the Barnes maze.

Importantly, we did not detect differences in motor function and overall activity in TIMP2 KO mice (Supplementary Fig. 3B–J). Motor coordination perturbations have been reported in TIMP2 mutant mice [13], so we subjected the mice to a wide battery of motor function, coordination, and activity assays, including rotarod, open field, wire testing, pole testing, and grip strength, none of which revealed changes in corresponding function between TIMP2 KO and WT mice (Supplementary Fig. 3B–I). We detected impaired clasping behavior in TIMP2 KO relative to WT mice (Supplementary Fig. 3J), as previously reported [13], but this form of motor coordination does not impact performance observed for TIMP2 KO mice in the wide range of hippocampus-dependent tasks utilized. No differences in anxiety-related behaviors in the open field were observed between WT and TIMP2 KO mice (Supplementary Fig. 3F). Collectively, these results indicate a critical role for TIMP2 in regulation of hippocampus-dependent memory.

### TIMP2 ablation leads to accumulation of ECM in the hippocampus

**Fig. 5:**
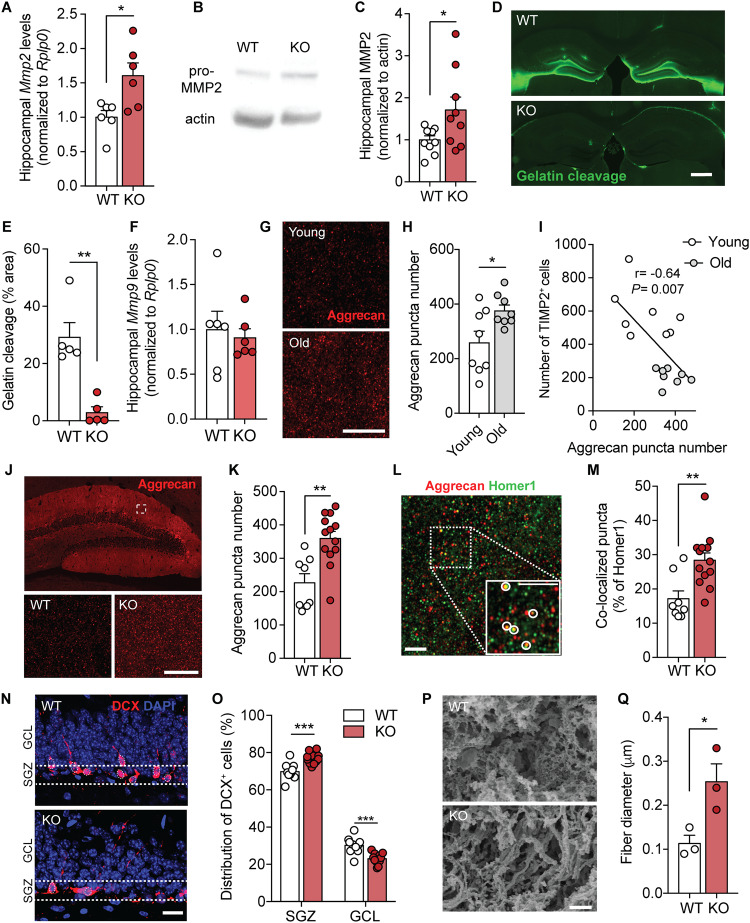
TIMP2 ablation leads to accumulation of ECM within the hippocampus. **A** *Mmp2* mRNA levels by qPCR from isolated hippocampus of WT and TIMP2 KO mice (2–3 months of age, *N* = 6 mice per group). **B** Immunoblotting of MMP2 from hippocampal lysates of WT and TIMP2 KO mice (2–3 months of age, *N* = 9 mice per group) with corresponding (**C**) quantification of MMP2 protein levels. **D** Representative images of fluorescence following DQ gelatin injection into 5-month-old WT and TIMP2 KO hippocampus with corresponding (**E**) quantification of fluorescence in hippocampus as percentage area (*N* = 5 mice per group; scale bar, 500 μm). **F** *Mmp9* mRNA levels by qPCR from isolated hippocampus of WT and TIMP2 KO mice (2–3 months of age, *N* = 6 mice per group). **G** Representative confocal microscopy images of aggrecan puncta in the molecular layer of the DG from young (3-month-old) and old (21-month-old) mice (*N* = 8 mice per group; scale bar, 5 μm), with corresponding (**H**) quantification of aggrecan puncta number. **I** Scatterplot of TIMP2^+^ cell number and aggrecan levels in young (3-month-old) and old (21-month-old) hippocampus. (*N* = 8 mice per group; Pearson correlation coefficient (r) = −0.64, *P* = 0.007). **J** Representative confocal microscopy images showing aggrecan puncta in the molecular layer of DG at low-magnification (top) and high-magnification (bottom) of analysis inset from WT and TIMP2 KO mice (2–3 months of age, *N* = 8–13 mice per group; scale bar, 5 μm) with corresponding (**K**) quantification of puncta. **L** Representative confocal microscopy image indicating colocalized puncta (see inset) with corresponding (**M**) quantification of aggrecan and homer1 co-localization in the molecular layer of DG from WT and TIMP2 KO mice (2–3 months of age, *N* = 8–13 mice per group; scale bar, 2.5 μm). **N** Representative images showing the migration of DCX^+^ cells in the SGZ and GCL of WT and TIMP2 KO mice (2–3 months of age, *N* = 11–12 mice per group, scale bar, 20 μm with corresponding (**O**) quantification of DCX^+^ cells distributed in the subgranular zone (SGZ) and granule cell layer (GCL) of DG. **P** Representative surface scanning electron microscopy (SEM) from DG of WT and TIMP2 KO mice (2–3 months of age, *N* = 3 mice per group, scale bar, 2 μm, with corresponding (**Q**) quantification of ECM microarchitecture in terms of fiber diameter measurements. Data are represented as mean ± SEM. Student’s *t* test for two-group comparisons. *P < 0.05, ** *P* < 0.01, ***P < 0.001. Data points represent individual mice.

We next investigated potential mechanisms that may account for how spine formation and adult DG neurogenesis are regulated by TIMP2. Outside the brain, TIMP2 has been linked to ECM degradation and remodeling through its canonical binding partner MMP2 [6, 7]. Brain ECM is dynamic, and accumulating evidence suggests a role for ECM and its components in the regulation of synaptic plasticity, including in spine remodeling [19, 34], adult neurogenesis [35], and cognition [35]. We first assessed the levels of pro-MMP2 in lysates of hippocampi from TIMP2 KO and WT mice. Interestingly, in the absence of TIMP2, we found significantly elevated levels of MMP2, both at the gene (Fig. 5A) and protein (Fig. 5B,C) levels, reflecting an accumulation in response to loss in TIMP2 signaling and pointing to a potential disruption in ECM turnover in the hippocampus. Since TIMP2 canonically facilitates conversion of pro-MMP2 to active MMP2 gelatinase [6], we next measured MMP2 activity by in vivo zymography [36]. A fluorescein-quenched gelatin substrate that fluoresces upon cleavage by active MMP2/9 was injected stereotaxically in the hippocampus, revealing high activity in the WT hippocampus that is lost with TIMP2 deletion (Fig. 5D, E). Together with our data showing that MMP9 is not significantly altered by TIMP2 deletion (Fig. 5F), our results argue that TIMP2 deletion results in aberrant MMP2 regulation.

Recent work indicates that brain ECM levels increase with age in parallel with age-dependent cognitive decline [37]. In the brain, chondroitin sulfate proteoglycans (CSPGs) are the major class of ECM proteins [38, 39]. The proteoglycan aggrecan, a core CSPG expressed in perineuronal nets in the brain, is highly enriched in the ECM of the DG [40] and has been linked to synaptic plasticity [41] and memory [42, 43]. Using super-resolution confocal imaging, we find that aggrecan is significantly increased in the hippocampus of aged mice (Fig. 5G, H), which is significantly correlated with the declining levels of hippocampal TIMP2 with age [4] (Fig. 5I). We therefore analyzed aggrecan distribution in the hippocampus of young WT and TIMP2 KO mice and found that TIMP2 KO mice exhibited a significant increase in the density of aggrecan puncta in the DG molecular layer (Fig. 5J, K), the same area where we observed impaired spine remodeling in TIMP2 KO relative to WT mice (Fig. 3). In support of this effect, we found that the intensity of *Wisteria floribunda* agglutinin (WFA)-positive perineuronal nets in the DG was similarly increased in the DG of TIMP2 KO compared to WT mice (Supplementary Fig. 4A, B).

To further determine if TIMP2 remodeling of the ECM could affect accumulation of ECM near synapses given the synaptic deficits we observed in these neurons, we analyzed aggrecan colocalization with the postsynaptic protein Homer1. We observed that the DG of TIMP2 KO mice exhibited significantly increased colocalization of aggrecan with Homer1 relative to that observed in WT DG (Fig. 5L, M). These data indicate that TIMP2 normally promotes ECM remodeling and limits its deposition near synapses.

We next further characterized the relationship between TIMP2’s effect on ECM and adult neurogenesis, considering the known roles of ECM and its related components in the regulation of proliferation and migration of newborn neurons (reviewed in [44]). To explore this possibility, we evaluated migration of newborn neurons toward the molecular layer by examining the distribution of DCX^+^ immature neuroblasts in the SGZ relative to that in the GCL of the DG [45]. We detected a significantly lower proportion of DCX^+^ immature neurons distributed in the GCL of TIMP2 KO mice relative to WT (Fig. 5N, O), reflecting a process whereby migration of newborn neurons within the DG is impaired in the absence of TIMP2. These data suggest that the absence of TIMP2 leads to a tighter ECM structural environment that acts to spatially restrict the migration of newborn neurons and plasticity of synapses, thus affecting overall adult neurogenesis and spine dynamics. In support of this concept, we measured structural changes in the ECM of TIMP2 KO mice by performing surface scanning electron microscopy (SEM) on decellularized brains isolated from TIMP2 KO and WT mice. Interestingly, we detected altered ECM microarchitecture in the form of thicker ECM fibers (Fig. 5P, Q) in hippocampi from TIMP2 KO compared to WT mice. Together, our results suggest that TIMP2 modulates the flexibility of the ECM within the hippocampus to regulate plasticity processes, including adult neurogenesis and spine plasticity.

### Neuronal TIMP2 regulates hippocampus-dependent cognitive function and adult neurogenesis

To gain further mechanistic insight into TIMP2’s role in the function of the hippocampus with increased cellular resolution, we generated *Timp2*-floxed (TIMP2^fl/fl^) mice using two-cell homologous recombination (2C-HR)-CRISPR/Cas9-based genome editing. We inserted two *loxP* sites flanking exon 2 of the *Timp2* gene transcript (Supplementary Fig. 5A, B) and then crossbred TIMP2^fl/fl^ mice with Syn^Cre/+^ mice (Supplementary Fig. 5C) to specifically excise *Timp2* in neurons. Immunoblotting against TIMP2 from isolated hippocampus lysates of Syn^Cre/+^; TIMP2^fl/fl^ or TIMP2^fl/fl^ control mice confirmed that conditional targeting of TIMP2 resulted in significantly reduced TIMP2 levels compared to TIMP2^fl/fl^ controls (Supplementary Fig. 5D, E). We performed further validation by confocal microscopy in the hippocampus of Syn^Cre/+^; TIMP2^fl/fl^ or TIMP2^fl/fl^ control mice to examine cells expressing TIMP2. Quantification of TIMP2^+^ NeuN^+^ cells in the hilus/DG, CA3 and CA1 regions revealed significant abrogation of TIMP2 in neurons upon its conditional deletion (Supplementary Fig. 5F,G). These data validate our model as a tool for addressing the role of neuronal TIMP2 in regulating the key cellular and behavioral phenotypes observed in the context of global TIMP2 deletion.

**Fig. 6:**
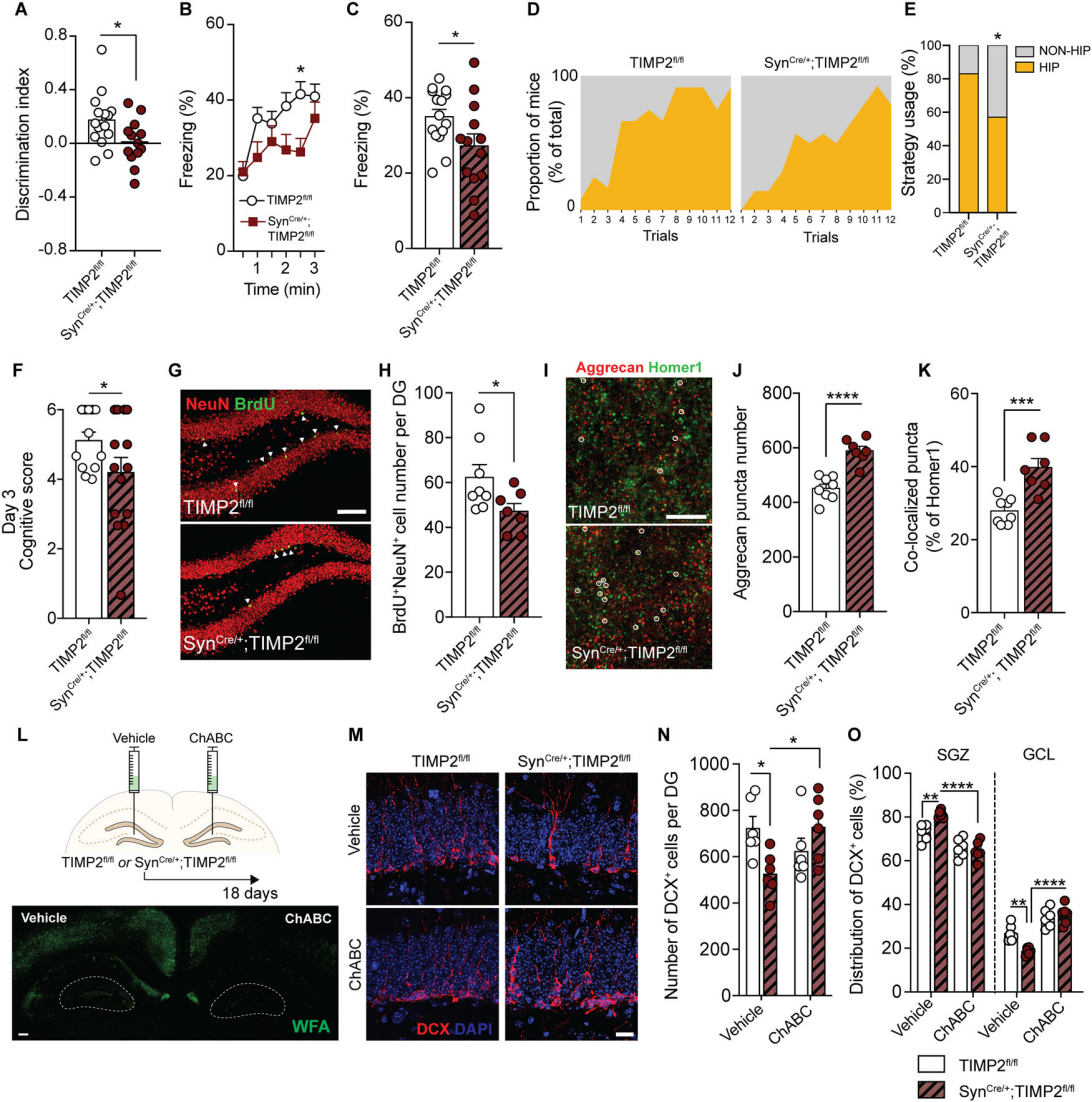
Neuronal TIMP2 regulates hippocampus-dependent cognitive function and adult neurogenesis. **A** Discrimination index for novel location recognition on day 2 and (**B**, **C**) contextual fear-conditioning freezing levels in TIMP2^fl/fl^ and Syn^Cre/+^; TIMP2^fl/fl^ mice (2–3 months of age, *N* = 13–15 mice per group). **D** Proportion of mice of each genotype using non-hippocampus-dependent (gray) and hippocampus-dependent (yellow) search strategies during the testing trials of TIMP2^fl/fl^ and Syn^Cre/+^; TIMP2^fl/fl^ mice in Barnes maze (2–3 months of age, *N* = 12–14 mice per group) with (**E**) quantification of the percentage of mice using these strategies on day 3. **F** Cognitive complexity scores for TIMP2^fl/fl^ and Syn^Cre/+^; TIMP2^fl/fl^ mice based on strategies used on day 3 of Barnes maze. **G** Representative confocal microscopy images of BrdU^+^ and NeuN^+^ cells in DG of TIMP2^fl/fl^ and Syn^Cre/+^; TIMP2^fl/fl^ mice (2–3 months of age, *N* = 7–8 mice per group, scale bar, 100 μm) with corresponding (**H**) quantification of BrdU^+^ NeuN^+^ newborn neurons in the DG. **I** Representative confocal microscopy images of aggrecan and homer1 puncta in molecular layer of DG of TIMP2^fl/fl^ and Syn^Cre/+^; TIMP2^fl/fl^ mice (*N* = 7–8 mice per group, scale bar, 5 μm) with corresponding (**J**) quantification of aggrecan puncta and (**K**) co-localized puncta of aggrecan and homer1, as indicated by overlaid circles. **L** Schematic depicting ChABC injection paradigm with representative WFA staining following ChABC digestion in DG; scale bar, 50 μm. **M** Representative confocal microscopy images of DCX^+^ cells in DG of TIMP2^fl/fl^ and Syn^Cre/+^; TIMP2^fl/fl^ mice injected with ChABC or vehicle (2–3 months of age, *N* = 6 mice per group; scale bar, 20 μm) with corresponding quantification of (**N**) number of DCX^+^ immature neuroblasts and (**O**) proportion of DCX^+^ cells in SGZ or GCL in DG of TIMP2^fl/fl^ and Syn^Cre/+^; TIMP2^fl/fl^ mice. Data are represented as mean ± SEM. Student’s *t* test for two-group comparisons in (**A–C**), (**H**), and (**J**, **K**), chi-square test in (**E**), nested *t*-test (**F**) with trial and mice as levels, and 2-way RM-ANOVA, Sidak’s post-hoc test in (**N**, **O**). *P < 0.05, ** *P* < 0.01, ***P < 0.001, ****P < 0.0001. Data points represent individual mice.

Given that we found that TIMP2 was expressed in neurons in the DG and present at significant levels in the extracellular space adjacent to these cells, we sought to determine whether the pool of neuronal TIMP2 is specifically involved in hippocampus-dependent memory. Using the Syn^Cre/+^; TIMP2^fl/fl^ mice we generated and validated (Supplementary Fig. 5), we created cohorts of Syn^Cre/+^; TIMP2^fl/fl^ mice and TIMP2^fl/fl^ control littermates to evaluate performance in spatial memory tasks. We found that Syn^Cre/+^; TIMP2^fl/fl^ mice exhibited significantly decreased preference for the displaced object 24 h after training in the testing phase compared to TIMP2^fl/fl^ controls, as indicated by a reduced discrimination index in these mice (Fig. 6A). Further assessment in the contextual portion of fear-conditioning revealed a significant reduction in freezing levels by Syn^Cre/+^; TIMP2^fl/fl^ mice compared to TIMP2^fl/fl^ controls, suggesting impaired contextual memory in the absence of neuronal TIMP2 (Fig. 6B, C). Cued freezing levels were indistinguishable between the groups of mice (Supplementary Fig. 6A). We then subjected the mice to Barnes maze testing to examine performance in search strategies to find the escape hole. Our search strategy analysis showed that a smaller proportion of Syn^Cre/+^; TIMP2^fl/fl^ mice used hippocampus-dependent strategies compared to TIMP2^fl/fl^ mice (Fig. 6D, E). Scoring their search strategy utilization by complexity shows that Syn^Cre/+^; TIMP2^fl/fl^ mice score lower than their TIMP2^fl/fl^ counterparts (Fig. 6F), suggesting TIMP2 from neurons is required for normal hippocampus-related memory and learning. Importantly, we did not detect differences in overall motor function in terms of coordination (Supplementary Fig. 6B, C), overall activity in the open field (Supplementary Fig. 6D, E), motor learning or grip strength (Supplementary Fig. 6G–I), and clasping behavior (Supplementary Fig. 6J) in Syn^Cre/+^; TIMP2^fl/fl^ mice relative to TIMP2^fl/fl^ mice. No differences in anxiety-related behaviors in the open field were observed between TIMP2^fl/fl^ and Syn^Cre/+^; TIMP2^fl/fl^ mice (Supplementary Fig. 6F).

To test whether the local pool of neuronal TIMP2 affects the survival of newly generated neurons, we analyzed the pool of BrdU^+^ NeuN^+^ cells after pulse-chase labeling with BrdU, as described earlier (Fig. 2B). Indeed, we observed that the deletion of TIMP2 within neurons resulted in a significant decrease in the number of BrdU^+^ NeuN^+^ newborn neurons compared to TIMP2^fl/fl^ controls (Fig. 6G, H). To probe potential mechanisms by which TIMP2 expressed from neurons affects hippocampus-dependent memory and adult neurogenesis, we next addressed ECM-related remodeling in the DG of Syn^Cre/+^; TIMP2^fl/fl^ mice. TIMP2 deletion in neurons in Syn^Cre/+^; TIMP2^fl/fl^ mice resulted in a significant increase in the density of aggrecan-positive puncta compared to TIMP2^fl/fl^ controls (Fig. 6I, J), as well as increased colocalization of aggrecan with Homer1 (Fig. 6I, K), consistent with an accumulation of ECM components adjacent to synapses, as observed earlier. Together, our results show that the pool of neuronal TIMP2 is necessary to promote ECM remodeling to enable synaptic plasticity and adult neurogenesis to facilitate normal hippocampus-dependent memory.

To determine the relationship between TIMP2’s effect on ECM and adult neurogenesis, we injected Chondroitinase ABC (ChABC) into the DG of TIMP2^fl/fl^ and Syn^Cre/+^; TIMP2^fl/fl^ mice, which targets ECM integrity via degradation of glycosaminoglycan side chains on CSPGs (Fig. 6L) [19, 46]. As expected, we first found that the number of DCX^+^ cells was significantly reduced in vehicle-treated DG of Syn^Cre/+^; TIMP2^fl/fl^ mice compared with vehicle-treated DG of TIMP2^fl/fl^ mice (Fig. 6M, N). ChABC treatment in Syn^Cre/+^; TIMP2^fl/fl^ mice rescued the number of DCX^+^ cells to vehicle levels seen in control mice (Fig. 6M, N). Strikingly, ChABC treatment in Syn^Cre/+^; TIMP2^fl/fl^ mice also rescued the deficit in migration of DCX^+^ cells from SGZ to GCL (Fig. 6M, O). Taken together, our results support a model in which ECM remodeling by TIMP2 regulates measures of hippocampal plasticity, including adult neurogenesis.

## Discussion

Our study illustrates how alterations in the ECM within the hippocampus by TIMP2 can directly regulate features of plasticity, including dendritic spine plasticity, adult neurogenesis, and hippocampus-dependent memory. We find that TIMP2 is highly expressed in hippocampal neurons and found at high levels in the brain extracellular space. Its removal induces structural synaptic changes and deficits in adult neurogenesis, as predicted from changes observed at the transcriptomic level, both of which result in impairments in hippocampus-dependent memory function. We demonstrate that TIMP2’s role in ECM remodeling is associated with changes in the flexibility of synaptic plasticity, impinging on processes like migration of new neurons. We generate and characterize a novel mouse model for conditional deletion of TIMP2 within neurons, highlighting molecular mechanisms through which neuronal TIMP2 promotes synaptic plasticity through regulation of the ECM.

The development and maintenance of synapses is critical in the establishment of functional neuronal circuits, and emerging evidence indicates a role for the ECM and its components in regulating synapse structure and morphology [44, 47, 48]. We used high-resolution imaging of labeled granule cells in the intact hippocampus of TIMP2 KO and WT mice to show that TIMP2 regulates dendritic spine density, with an increase in thin spines and a reduction in more mature mushroom spines [28] in KO mice. The timecourse over which TIMP2 is targeted in our study overlaps with both early and ongoing processes of spinogenesis and spine maturation, perhaps affecting spine dynamics in an interrelated fashion, including for thin spines and mushroom spines, that could potentially influence memory. Future work may dissect the precise timescale in which TIMP2 acts on specific classes of spines. Moreover, when TIMP2 is targeted either globally or specifically within neurons, ECM components like aggrecan accumulate around hippocampal synapses, and the ECM is more dense, as shown by our SEM data, suggesting that TIMP2 acts directly within the hippocampus to modulate plasticity in a spatial sense. In agreement with its role in modulating plasticity, we also find that TIMP2 regulates the process of adult neurogenesis. While immature neuroblasts were unaltered in mice with systemic modulation of TIMP2 [4], presumably due to the window over which TIMP2 was modulated being too short to reflect changes in DCX^+^ cells, we now show that removal of TIMP2, both globally and from neurons in the hippocampus, results in disruption in neural progenitor proliferation and maturation. This is in line with in vitro evidence indicating that TIMP2 was significantly upregulated upon neuronal differentiation [49] and may be involved in the process [12]. Intriguingly, we observed that TIMP2 deletion affects migration of immature DCX^+^ neurons toward the molecular layer, arguing for a role of ECM in regulating neuronal migration [50]. While ECM molecules have been documented to regulate neurogenesis [44], the mechanisms are unclear. CSPGs surround neural stem cells and progenitors in the hippocampus and have been shown to support neural progenitor proliferation [35] and migration [51], suggesting that healthy regulation of the ECM supports normal proliferation and migration of adult neurogenesis and in an interrelated fashion. ECM modulators should be examined in other brain regions that may depend on ECM turnover for region-relevant processes.

TIMP2 is recognized for its unique dual role in regulating both the activation of pro-MMP2 to facilitate active MMP2 and the inhibition of MMP2 activity under certain conditions to regulate ECM remodeling [6, 7]. We observed that deletion of TIMP2 is associated with increased *Mmp2* and pro-MMP2 levels in hippocampus with concomitant loss of MMP2 activation, reflecting compensatory responses to reduced TIMP2, as described outside the brain [6, 7]. Accumulated pro-MMP2 translates to less remodeling of the ECM and its resulting accumulation around cells of the DG neurogenic niche. A proposed mechanism of spine stabilization and remodeling by ECM includes a structural-spatial restriction mechanism in which ECM components, including CSPGs, form a matrix around dendritic spines to provide extracellular rigidity and physically restrict spine motion [52]. MMPs themselves can modulate structural plasticity by loosening the ECM structure, allowing for a more permissive environment [44]. Stiffness of the surrounding environment determined by ECM complexity has been implicated in regulating cell migration [53, 54], including for neural stem cells in vitro [55–57]. This modulation has been proposed to be among fundamental cell fate-determining factors in neural development and neurological disorders [58–60]. Our results argue that ECM accumulation in the absence of TIMP2 leads to a denser network that physically impedes dendritic spine turnover, as well as proliferation and migration during adult neurogenesis.

In line with reports linking disrupted synaptic plasticity and neurogenesis with cognitive dysfunction [44], we show that TIMP2-related changes in hippocampal plasticity are associated with altered memory. Both global and neuron-specific targeting of TIMP2 levels resulted in hippocampus-dependent memory deficits, though future work overexpressing TIMP2 may clarify these effects; previous supplementation work in aged mice is consistent with a role for TIMP2 in regulating hippocampus-dependent memory [4]. Our results reinforce an exciting link among TIMP2, synaptic plasticity, and memory in the healthy hippocampus through regulating spine dynamics and neurogenesis. The cognitive phenotypes described here cannot be attributed to TIMP2’s role in other brain regions related to motor function, though earlier work in younger mice described some deficits in coordination upon TIMP2 deletion [13], primarily related to cerebellar alterations [13]. We show that TIMP2 deletion is associated with changes in MMP2. TIMP2 may interact with other MMPs and binding partners (e.g., alpha3beta1 integrin [12]) to mediate its diverse biological functions and in a manner that varies depending on in vivo or in vitro contexts. Moreover, while we mostly find TIMP2 expressed in neurons in our study, its expression in other cell types may be important in certain pathological contexts [61] to be explored further, especially as a role for microglia in ECM remodeling to promote synapse plasticity in the hippocampus was recently described [19]. Additional insights may come from investigations of TIMP2 regulation at varying timepoints across lifespan in the CNS and the extent to which the effects are conserved across neuronal subtypes.

Dysregulation of the ECM is implicated in cognitive dysfunction that occurs in aging [62] and in disease [63]. The progressive decline in TIMP2 expression with age [4] occurs in parallel with deficits in memory [64] and ECM accumulation, as demonstrated here, while systemic treatment with TIMP2 revitalizes hippocampus-related function in aged mice [4]. Moreover, ECM clearance is beneficial in mouse models of Alzheimer’s disease pathology [65], and it has been increasingly appreciated that adult neurogenesis in the human DG is dysregulated in Alzheimer’s disease [66]. Defining molecular mechanisms accounting for how modulators of the ECM, including TIMP2, regulate the healthy brain may lead to development of novel approaches for cognitive repair in contexts of aging or neurodegeneration.

### Supplementary information

Supplementary Information
