# Supplementary material for: Neuronal TIMP2 regulates hippocampus-dependent plasticity and extracellular matrix complexity
Source: Mol Psychiatry. 2023 Nov 2;28(9):3943–54. doi: 10.1038/s41380-023-02296-5 (PMC10730400; doi:10.1038/s41380-023-02296-5)
Supplement: Supplementary file 1 — Supplementary Information [file 41380_2023_2296_MOESM1_ESM.docx]

**Supplementary Information, *Materials and Methods and Supplementary Figures***

**Materials and methods**

**Generation of TIMP2-floxed mice**

To achieve conditional deletion of *Timp2*, a loxP-flanked *Timp2* allele (TIMP2^fl/fl^) was generated by the two-cell homologous recombination (2C-HR)-CRISPR/Cas9-based genome editing method, as previously described^1^. Briefly, two loxP sites were inserted into the *Timp2* gene flanking exon 2, with homologous arms (HA) on each side. Two point-mutations (C928->A near the end of the left arm and G1795->T at the 5’ junction of the right arm) were introduced to disrupt the PAM sequence to avoid re-cutting of the recombined allele. Three nucleotides adjacent to each loxP site were altered to create restriction sites for diagnostic purposes: XbaI near the upstream loxP, and HindIII near the downstream loxP site. The two-cell stage embryo microinjection procedure was performed as in^1^, using Cas9-mSA mRNA, biotinylated double-stranded PCR product (1016 nts), and pre-assembled ctRNA prepared accordingly^1^. F_0_ mice were screened by sequencing from TOPO cloned PCR product, using tail genomic DNA, amplified from the TIMP2 target region, and using the following primers: FW-AGCGACCGATAAGCAGGAAG, Rev-CACTAGCAGACACCACCACA. Identified founders were crossed with wild-type (WT) C57BL/6J mice, purchased from The Jackson Laboratory, to produce heterozygous mice.

To achieve neuron-specific *Timp2* deletion, neuron-specific heterozygous Synapsin (Syn)-Cre (Syn^Cre/+^) transgenic mice^2^ (JAX stock #003966) were crossbred with TIMP2^fl/fl^ mice to generate Syn^Cre/+^; TIMP2^fl/+^ progeny. The F_1_ mice were then crossed to generate Syn^Cre/+^; TIMP2^fl/fl^ and TIMP2^fl/fl^ littermate controls. Cre expression was maintained in the female mice for breeding^2^. All animals were maintained on a C57Bl/6J background.

**Genotyping**

Genotyping of TIMP2^fl/fl^ mice was performed using the following primers: *Primer 1*: 5’- TGACCCCTCTGCTCTAGTCC, *Primer 2:* 5’-GCTCCGTCTCTTCGTCCATC (**Figure 6A**). Genomic DNA was extracted from the tail using the QIAamp Fast DNA Tissue Kit (Qiagen). PCR was performed in a total volume of 50 µl at 94°C for 2 min, 94°C for 30 s, 61°C for 30 s, 68°C for 2 min and 15 sec for 10 cycles, then 94°C for 30 s, 56°C for 30 s, 68°C for 2 min and 15 s for 25 cycles, and ending at 72°C for 5 min using Prime STAR (#R050A, TaKaRa, Shiga, Japan). For loxP site verification based on restriction digest, the PCR product (2.1 kb) was digested with XbaI and HindIII restriction enzymes (New England Biolabs, Ipswich, MA, USA) at 37°C for 1 hour, followed by 10 min at 65°C for heat inactivation. Digested PCR products were run on an agarose gel and imaged for the identification of founders: HindIII (WT: 2133 bp; upstream loxP positive: 829 bp and 1,304 bp); Xba1 (WT: 2133 bp; downstream loxP positive: 566 bp and 1,567 bp).

**In vivo microdialysis**

For guide cannula implantation into the hippocampus, mice were anesthetized with 2% isoflurane and head was shaved prior to being fixed in a stereotaxic apparatus (Stoelting Co., Wood Dale, IL, USA). After preparing skin with 70% ethanol and povidone-iodine, an anterior-to-posterior incision was made along the midline of the head to expose the skull. Skull position was leveled to within 0.1 mm along the bregma-lambda axis, and at 2.2 mm to the left and right of midline on the lateral axis. Holes were drilled to target the left hippocampus using the stereotaxic coordinates: bregma -3.1 mm, 2.5 mm lateral. A second hole was made diagonally to the first, to serve as an anchoring position with a bone screw. To target caudal hippocampus, AtomosLM Guide Cannula (PEG-12, Eicom, London, UK) was inserted at a 12° angle, 1.2 mm below the dura mater. Dental cement was applied to secure the cannula, and the skin was secured using a surgical adhesive glue. An AtmosLM Dummy Cannula (PED-12, Eicom) was inserted into the guide cannula and secured with a plastic cap nut. Animals were then placed in a clean cage on a heating pad and allowed to recover from surgery.

Approximately 12 hours following surgery, mice underwent 1000-kDa *in vivo* microdialysis. The inlet port of AtmosLM Microdialysis Probe (PEP-12-02, Emicon) was connected to a syringe pump (kdScientific, Holliston, MA, USA) perfusing artificial cerebrospinal fluid (aCSF), in mM: 1.3 CaCl_2_, 1.2 MgSO_4_, 3 KCl, 0.4 KH_2_PO_4_, 25 NaHCO_3_, 122 NaCl, pH 7.35, at a rate of 1.2 μl/min^3^. The outlet of the probe was connected to the peristaltic pump (MAB 20, SciPro, New York, NY, USA), which was calibrated to obtain a pull rate between 1-1.1 μl/min. After the probe was connected to the push-pull mechanism, the mice were briefly anesthetized with isoflurane to allow for the removal of the dummy cannula and insertion of the probe into the hippocampus. The probe was secured with a cap nut, and a plastic collar was loosely placed around the neck of the mouse. Mice were then moved to a Raturn (Stand-Alone Raturn System, BASi,
West Lafayette, IN, USA) and tethered by the collar to avoid tangling microdialysis tubing (FEP tubing 0.65 mm OD x 0.12 mm ID, BASi), while allowing for free movement of the mouse during the sample collection period. Samples were collected hourly in a refrigerated fraction collector (MAB 85 Fraction Collector, SciPro) and frozen at −80°C after collection. The procedure was performed under constant light conditions, and food and water were provided *ad libitum*.

**Bulk RNA-sequencing**

RNA-sequencing was performed on hippocampi dissected from WT and TIMP2 KO male and female mice. Mice heterozygous for TIMP2 were not used for analysis. Dissected hippocampi were preserved in RNAlater (Invitrogen) overnight at 4°C before storing at −80°C until use. RNA was extracted using the RNeasy Mini Kit (Qiagen), according to the manufacturer’s instructions. RNA quality was measured using the Agilent TapeStation Bioanalyzer (Agilent Technologies), and all samples exhibited RNA Integrity Number (RIN) > 8. cDNA libraries were prepared with poly(A) selection and sequenced using Illumina Hiseq (2x150bp paired-end) (Genewiz). At least 25 M clean reads were generated from each sample. The reads were mapped to the *Mus musculus* GRCm38 reference genome available on ENSEMBL, using STAR aligner (v.2.5.2b). After extraction of gene hit counts, DESeq2 was used for downstream differential expression analysis. The Wald test was used to generate *P*-values and log2 fold changes. Differentially expressed genes (DEGs; nominal *P*<0.05*)* were used for Gene Set Enrichment Analysis (GSEA; https://www.gsea-msigdb.org/gsea/index.jsp; last accessed Spring 2023). Volcano plot was generated with R (version 4.1.2). RNA-seq data files are available as GEO accession GSE223188.

**Weighted Gene Correlation Network Analysis (WGCNA)**

**The R package WGCNA (v. 1.71-6) was used to construct scale-free co-expression networks to identify modules with coordinated expression patterns according to TIMP2 genotype from expression values adjusted for batch effects as significant sources of variation (isolation/harvest date). Soft powers were used to calculate an adjacency matrix where the scale-free topology index reached 0.90 and the mean connectivity approached 0. A topological overlap matrix (TOM) was then created by transforming this adjacency matrix. The default parameters for signed networks were used with the customization: soft threshold power = 14, minimum module size = 30, cutting height = 0.99, merge cut height = 0.25, and deepSplit = 2. Distinct color identifiers were used to assign all genes to modules. The “grey” module was used to group genes that did not meet the criteria for module assignment. Modules from co-expressed genes were then used to calculate module eigengenes. Significant modules (*P*<0.05) were further analyzed by GSEA to examine gene ontology enrichment (**last accessed Spring 2023**).**

***In vivo* zymography and ChABC injections**

For stereotaxic procedures to bilaterally inject *in vivo* zymography or ChABC reagents into adult hippocampus, mice were anesthetized with 2% isoflurane, headfixed with a stereotaxic frame (Stoelting Co., Instrument, Word Dale, IL, USA), and treated with ophthalmic eye ointment. Fur was shaved and the incision site was sterilized with 70% ethanol and Betadine before surgical procedures. For *in vivo* zymography, holes were drilled bilaterally in the skull and 1.5 μl of DQ_TM_ Gelatin from pig skin, fluorescein conjugate (ThermoScientific, #D12054) was infused (2.0 mm AP, ± 1. 5 mm ML, 1.8 mm DV) at a rate of 0.2 μl/min, using a 5 μl-Hamilton syringe controlled by an infusion pump (KdScientific), according to a similar method^4^. Twenty minutes following removal of the injector, mice were anesthetized with a cocktail of ketamine (90 mg/kg) and xylazine (10 mg/kg) and transcardially perfused with ice-cold 0.9% saline, and then brains were collected and processed for imaging. For injections with Chondroitinase ABC (Sigma), 1 μl of 40 U/mL was unilaterally injected (2.0 mm AP, 1.5 mm ML, 2.0 mm DV) at a rate of 0.2 μL/min; contralateral site was injected with vehicle control. Body temperature for both procedures was maintained throughout surgery using a heating pad, and mice were allowed to recover on a heat pad before being returned to their cage.

**Immunohistochemistry and microscopy**

For all immunohistochemistry experiments, mice were anesthetized with a cocktail of ketamine (90 mg/kg) and xylazine (10 mg/kg) and transcardially perfused with ice-cold 0.9% saline. Brains were dissected, and hemibrains isolated for immunohistochemistry were postfixed in 4% paraformaldehyde (PFA) for 48 hours at 4°C before preservation in 30% sucrose (Sigma, St. Louis, MO, USA) solution in phosphate-buffered saline (PBS). Hemibrains were sectioned coronally at 40-µm thickness on a freezing-sliding microtome (SM2010R, Leica, Deer Park, IL, USA), and sections were stored in cryoprotective medium at −20°C.

For immunohistochemistry, free-floating brain sections were permeabilized with Tris-buffer saline (TBS) plus 0.05% Tween-20 (TBST) and incubated in a blocking solution consisting of 10% normal goat (#S-1000, Vector Laboratories, Newark, CA, USA) or donkey serum (#017-000-121, Jackson ImmunoResearch, West Grove, PA, USA) in TBST. Sections were then incubated with primary antibodies in 2% goat or donkey serum in TBST overnight at 4°C. Sections were incubated with primary antibodies, including the following: TIMP2 (1:100; #AF971, R&D Systems, Minneapolis, MN, USA), NeuN (1:200; #MAB377, Millipore, Burlington, MA, USA), doublecortin (DCX; 1:200; #4604S, Cell Signaling, Danvers, MA, USA), Sox2 (1:200; #sc365823, Santa Cruz, Dallas, TX, USA), Ki67 (1:200; #14-5698-82, Thermo Fisher, Waltham, MA, USA), aggrecan (1:500; #AB1031, Millipore), Homer1 (1:500; #160006, Synaptic Systems, Göttingen, Germany), Biotin Lectin from *Wisteria floribunda* (WFA) (1:1000; Millipore, #L1516). The next day, sections were washed in TBST three times for 5 min each and incubated with Alexa Fluor-conjugated secondary antibodies at 1:200 in TBST for 1 hour at RT. Secondary antibodies used were: Alexa Fluor 594-labeled donkey anti-rabbit (#A-21206), Alexa Fluor 594-labeled donkey anti-mouse (#A-21203), Alexa Fluor 488-donkey anti-rat (#A-21208), Alexa Fluor 488-donkey anti-goat (#A-11055), Alexa Fluor 647-donkey anti-mouse (#A-31571), Alexa Fluor 594-goat anti-rabbit (#A-11012), Alexa Fluor 5488-goat anti-chicken (#A-11039), all from ThermoFisher. Brain sections were then washed, stained with 4′,6-diamidino-2-phenylindole (DAPI, Sigma) for 15 min to visualize nuclei, mounted and coverslipped using Prolong Gold (Life Technologies) and dried overnight before imaging. For BrdU staining, brain sections were pre-treated with 3M HCl (Thermo Fisher) for 30 min at 37°C before overnight incubation with primary antibody anti-BrdU (1:500; #ab6326, Abcam, Cambridge, UK) at 4°C. Secondary staining and mounting were performed as described above.

Image processing was performed using an LSM 780 confocal microscope (Zeiss, Jena, Germany) using 40x/1.4 Oil DIC objective. Four equally-spaced sections per mouse (rostral through the caudal extent of the DG) were used to count the total number of positive cells within the DG (subgranular zone and hilar subregions) of the hippocampus, according to stereological principles. Quantification of fluorescence resulting from DQ gelatin cleavage was performed from thresholded DG area occupied by fluorescence (% area). WFA intensity was measured as the mean fluorescent intensities of 6 randomly chosen regions of interest (ROIs), each at 50 µm × 50 µm in area, within the DG molecular layer in each section. Doublecortin migration within the DG was quantified as proportion of DCX^+^ cells localized in the SGZ or GCL using the method defined in *Jiang* et al.^5^, calculated by normalizing the number of DCX^+^ in the SGZ or GCL to the total number of DCX^+^ cells.

Analyses were performed using FIJI in a blinded fashion, according to similar methods^6^.

**Aggrecan and Homer1 puncta quantification**

Quantification of the number of puncta by super-resolution microscopy proceeded similar to the method described previously^7^. Briefly, images were acquired by confocal microscopy imaging using an LSM 880 with AiryScan in super-resolution mode (Zeiss) set with a 63X/1.4 Oil DIC objective with 5X optical zoom. Using consistent laser strength, gain, and digital offset settings across all sections/experiments, Z stacks were acquired with steps less than the optical slices acquired. Using Zen software (Zeiss), a setting of 6 (“optimal”) was used during AiryScan processing. Aggrecan puncta were quantified using the Puncta Analyzer plugin^8^ in ImageJ, and thresholding was applied equally across images. For colocalization of co-stained Aggrecan and Homer1 puncta, images were analyzed with the Puncta Analyzer plugin with a minimum pixel specification of 4. Three images in the molecular layer of the DG were averaged per mouse for analysis.

**Dendritic spine analysis by iontophoretic dye injections**

*Tissue processing.* Mice were anesthetized using 15% chloral hydrate and transcardially perfused with 1% PFA in phosphate buffer (PB, pH 7.4) for 2 min, followed by 4% PFA with 0.125% glutaraldehyde in PB for 10 min at a rate of 5 ml/min. Brains were dissected and postfixed overnight at 4°C in the same fixative, and then transferred to PBS with 0.1% sodium azide at 4°C until sectioned. Brains were hemisected, and the right hemisphere was cut into 200 µm-thick sections using a vibratome (VT1000S, Leica).

*Intracellular dye injection and confocal imaging*. Coronal sections were incubated in 250 ng/ml DAPI for 5 min to enable identification of the DG. Sections were mounted on nitrocellulose membrane filters, immersed in ice-cold PB, and DG granule cells were iontophoretically injected with 5% Lucifer Yellow (Invitrogen, Waltham, MA, USA) in distilled water under a direct current of 3-8 nA until the dye filled the distal ends of the dendrites. Six to eleven neurons were injected per section and neurons selected for injection were spaced to avoid overlapping of dendrites. Sections were mounted with Fluoromount-G (#0100-01, Southern Biotech, Birmingham, AL, USA) between spacers placed on gelatin-coated glass slides (#22-214-320, Thermo Fisher).

To select dendritic segments for imaging on a Zeiss LSM 780 confocal microscope (Zeiss), whole cells at the suprapyramidal blade of the DG were first imaged using a 20x/0.8 M27 Plan-Apochromat objective, using an Ar/Kr laser at an excitation wavelength of 488 nm. Confocal stacks were acquired at 512 x 512-pixel resolution with a Z-step of 1 µm, a pinhole setting of 1 Airy Unit, and optimal settings for gain and offset. Basal dendritic segments at 50 µm from the soma were selected for high-resolution imaging according to the following criteria: not a primary dendrite, no overlap with other dendrites or branching that would obscure spines, not too deep in the section, and parallel or at acute angles to the coronal plane. Dendritic segments were imaged using a 100x/1.46 Oil DIC M27 Plan-Apochromat objective, and stacks were acquired at 512 x 512-pixel resolution with a Z-step of 0.1 µm, optical zoom of 3.3x, a pinhole setting of 1 Airy Unit, and optimal settings for gain and offset. The stacks were imaged with approximately 1 µm above and below the segment to fully include all spines. Three z-stacks were imaged from each neuron. Confocal stacks were deconvolved using an iterative blind deconvolution algorithm (AutoQuant X, vX3.0.1, MediaCybernetics, Rockville, MD, USA).

*Spine reconstruction and analysis.* Deconvolved stacks were analyzed using Neurolucida 360 (v2019.2.1; MBF Bioscience, Williston, VT, USA) for semi-automated reconstruction of dendrites and spines to determine spine density and morphology. Spines were classified as stubby, thin, mushroom, and filopodia, based on their morphologies, according to previous work^9^. 4-5 mice per genotype, 6 neurons per mouse, and 3 dendrites per neuron were analyzed, and violin plots were used to better visualize the distribution of individual data points.

#### **Scanning electron microscopy (SEM)**

#### Following ketamine/xylazine anesthesia and transcardial perfusion with PBS, 2-mm thick brain slices containing the hippocampus were decellularized, following previously described methods^10^. SEM methods were adapted from a previous study^11^. Decellularized brain slices were fixed in 4% PFA with 2% glutaraldehyde in 0.1M sodium cacodylate buffer (pH 7.4) for 24 hours at 4°C. Samples were then briefly rinsed in the same buffer before post-staining with 1% OsO4 for 1 hour. OsO_4_-treated samples were rinsed in water and gradually dehydrated in increasing concentrations of ethanol (50, 70, 90 100, and 100%, 10 min each). Samples were then stacked horizontally onto wire mesh dividers to keep them flattened and critical point-dried with liquid CO_2_. Dried samples were mounted onto Aluminum SEM stubs using conductive copper tape and sputter-coated before imaging with a Zeiss Supra 55 VP FESEM using InLens SE detection at 5 kV operating voltage (Zeiss Microscopy Inc). Fiber diameters were analyzed using Image J (Bethesda, USA).

**Behavior**

All behavioral analyses were performed during the 0700–1900 light cycle. For all behavioral experiments, age- (2-3-month-old) and sex-matched mice with corresponding littermate control animals were used.

*Novel location recognition.* Hippocampus-dependent memory was assessed using the novel location recognition as previously described^6^. On day 1, the training day, mice were habituated to the open-field arena for 6 min, an arena that contained wall-mounted visual cues. Mice were exposed to three consecutive trials of 6 min each, during which they explored two different objects in fixed positions. On day 2, the testing day, mice explored the same arena as on the training day but with one object displaced to a novel position. Time spent exploring each object was manually scored in a blinded fashion to assess the discrimination index for the novel location as the displaced object interaction time/total interaction time of both objects.

*Contextual fear-conditioning.* Fear-conditioning experiments were performed as previously described^6^ to assess hippocampus-dependent memory. Briefly, mice were trained to associate the cage context with an aversive stimulus (light foot-shock; Ugo Basile). On the first day, training parameters included two periods of 30 s consisting of a paired cue light and a tone of 1,000-Hz, followed by a light foot-shock (2 s, 0.5 mA) separated by a 180-s interval. Chambers were cleaned with 70% ethanol between experiments by the experimenter. Twenty-four hours later, mice were re-exposed to the same context for 3 min, and freezing levels (contextual) were measured. Two hours later, for the cued task, mice were placed in a novel context and exposed to the same tone and cue light from day 1 (training) after 120 s of exploration. Freezing levels were analyzed for pre- and post-cue phases. Freezing levels were measured using EthoVision XT system software (v14.0.1319, Noldus).

*Barnes maze.* Barnes maze testing was performed similarly to that previously described^6^. A large circular maze containing 40 holes was centered over a pedestal and elevated approximately 40 cm above the floor with a video recording device mounted directly above. The escape hole consisted of a PVC box similar in texture and color to the surface of the maze. Distinct visual cues were placed at four equally spaced points around the maze to serve as spatial navigation cues as the mice navigate the task. The task proceeded over 4 days, with four trials on each day for each mouse. With overhead illumination and a persistent 2 kHz tone, mice were given 90 s for each trial to identify the escape hole by jumping in or identifying the hole with extended/overhead pokes. If mice failed to find the escape hole within 90 s, they were gently guided by light tapping/directing towards the escape hole and scored with 90 s. The escape hole position was fixed within a day but changed for each successive day of testing. For each trial within a day, the starting location for the mouse was rotated relative to the escape hole position. Data collection and analyses were performed using EthoVision video-tracking system.

Search strategy classification was manually performed and based on methods adapted from previous work^12^, with data collected by EthoVision video-tracking system software. Search strategies were coded according to the following: (i) localized - minimal movement from the starting position; (ii) serial - sequential nose points within the outer ring area; (iii) random - unorganized searching; (iv) scanning - arc-like trajectory, without nose points; (v) focal - direct search within a maze quadrant; (vi) focal missense - direct search within a maze quadrant from previous trials; (vii) targeted - direct movement to hole with deviations in the trajectory direction; (viii) direct - no shifts in the trajectory direction with single nose point to target hole. For strategy analysis, each related strategy was categorized into non-hippocampus-dependent strategies comprising localized, serial, random, and scanning strategies, and hippocampus-dependent strategies that included focal, focal missense, targeted and direct strategies. Cognitive performance of each trial on day 3 was scored such that cognitive strategies received higher scores according to the following scale, accounting for high cognitive complexity, with a separation of 1 point between non-hippocampus-dependent and hippocampus-dependent strategies: localized = 0, serial = 1, random = 2, scanning = 3, focal = 5, focal missense = 5, targeted = 6, direct = 6. Focal and focal missense strategies are assigned a score of 5, as both are goal-directed strategies used without achieving the final target. Target and direct strategies were assigned score of 6 since both are goal-directed strategies with successful achievement of the final target.

*Open field*. The open field test was used to evaluate locomotion and exploratory behavior, and anxiety-like behavior in the center of the arena. The apparatus consisted of a brightly illuminated square arena of 50 x 50 cm enclosed by walls 45 cm high. Mice were placed individually in the center of the open field arena, and their movement was traced for 6 min. The resulting data was analyzed using EthoVision software, considering two previously defined areas: a central and an outer area. Due to the thigmotaxic exploratory activity of rodents, the ratio between the time spent in the center and in the periphery of the open field arena can reflect anxiety-like behaviors. Distance traveled, average velocity, and time spent in each of the zones were recorded and analyzed.

*Rotarod*. Balance and motor function was measured using the fixed and standard accelerated rotarod test (Med Associates). The protocol consisted of a first day of training at a constant speed (4 rpm) for a maximum of 2 min in three trials, with a 2-min interval between each trial. On day 2, animals were tested for each of 3 different fixed speeds over a range of 4 to 40 rpm for a maximum of 60 sec each in three trials, with a 2-min interval between each trial. For the acceleration test, animals were placed on the rod rotating at a constant speed (4 rpm), then the rod started to accelerate continuously from 4 to 40 rpm over 5 min, in three trials with a 5-min interval between each trial. The latency to fall off the rotarod was recorded for each trial, and data from three trials were averaged for each mouse.

*Wire grip test.* Motor coordination and strength was measured by the wire grip task. Each mouse was placed on a wire cage top, which was slowly inverted and suspended at approximately 30 cm above a mouse cage. The latency of time until the mouse fell off the wire was recorded in three trials. Mice that did not fall within the 60-s trial period were removed and assigned a maximal time of 60 s. Data from three trials were averaged for each mouse.

*Clasping.* Mice were suspended by their tail, and the extent of hindlimb clasping was observed for 15 s. Clasping scoring was based on a previously described method^13^. Briefly, if both hindlimbs were splayed outward away from the abdomen with splayed toes, a score of 0 was given. If one hindlimb was retracted or both hindlimbs were partially retracted toward the abdomen without touching it, and the toes were splayed, a score of 1 was assigned. If both hindlimbs were partially retracted toward the abdomen and were touching the abdomen without touching each other, a score of 2 was given. If both hindlimbs were fully clasped and touching the abdomen, a score of 3 was assigned.

*Grip strength.* For the grip strength test, forelimb, hindlimb, and 4-limbs grip strength was evaluated using a grip strength meter (Bioseb). Animals were held by the tail and allowed to place forelimbs on the bar, or they were restrained to place hindlimbs on the bar connected to the force sensor. Mice were slowly pulled away until they released the bar, and the maximal grip force measured during the pulling session was recorded. For the 4-limb measurement, using a mesh pull bar attachment, animals were handled by the tail so that all the paws latched onto the mesh pull the bar together. The mice were slowly and steadily pulled away from the apparatus exactly parallel from the countertop until paws were released from the mesh. A modified version of the forelimb grip strength test was performed as previously described^14^. Briefly, the meter was rotated vertically and the measurement procedure was identical to the conventional test, except for the direction in which the mouse’s tail was pulled. Each animal’s grip strength (in *g*) was recorded, and data from five trials were collected.

*Pole test.* Motor coordination and balance with striatal involvement was assessed with the vertical pole test, which consisted of a plastic pole (50 cm high, diameter 1 cm) placed vertically on a mouse cage. Mice were individually placed at the top of the pole facing upward by their front paws on the pole, and the time taken to turn around and climb down the pole was recorded. A maximum time of 120 s was given to complete the task. If the mouse fell from the top of the pole, a time of 120 s was recorded. Each mouse underwent three trials, and the pole was cleaned with 70% ethanol between animals. Data from three trials were averaged for each mouse.

**qPCR**

After rapid dissection of the hippocampus, tissue was preserved in RNAlater (Invitrogen) overnight at 4°C before storing at −80°C until use. Hippocampal RNA was extracted according to the manufacturer’s instructions using a RNeasy Mini Kit (Qiagen). Quality and concentration of total RNA were measured on the Nanodrop 8000 (Thermo Fisher). cDNA was subsequently synthesized with SuperScript III (Invitrogen), following the manufacturer’s recommendations. Samples were mixed with SYBR Green master mix (Thermo Fisher) and primers for *Mmp2* (FW: 5'-CAAGTTCCCCGGCGATGTC-3'; REV: 5'-TTCTGGTCAAGGTCACCTGTC-3') or *Mmp9* (FW: 5'-CTGGACAGCCAGACACTAAAG-3'; REV: 5'-CTCGCGGCAAGTCTTCAGAG-3') before loading as technical replicates for qPCR on a QuantStudio 7 Flex Real-Time PCR System (Thermo Fisher). Cycle counts for mRNA quantification were normalized to *Rplp0* (FW: 5'-AGATTCGGGATATGCTGTTGGC-3'; REV: 5'-CCAGTTGGTAACAATGCCATGT-3'). The ΔΔCT method was used to provide gene expression values, and expression levels were normalized to *Rplp0*.

**Western blotting**

For Western blotting of MMP2 or TIMP2 within hippocampus, dissected hippocampi were manually homogenized with 60 strokes in RIPA lysis buffer (Thermo Fisher), with protease inhibitors (Roche), before centrifugation at 20,000g for 25 min at 4°C. Protein concentration of the supernatant was determined using the BCA kit (Thermo Fisher). Samples with equal amounts of protein were separated on 4-12% NuPAGE Bis-Tris precast denaturing gels (Invitrogen) and transferred onto nitrocellulose membranes (BioRad, Hercules, CA, USA) at 125 V for 90 min. Membranes were blocked with 3% milk-TBST (0.125% Tween) for 1 hour at room temperature and then probed with primary antibodies diluted in 3% milk-TBST solution overnight at 4°C: anti-TIMP2 (1:500; #D18B7, Cell Signaling), anti-MMP2 (1:1000; #ab86607, Abcam), and anti-actin (1:10000; #A5060, Sigma). Membranes were washed and probed with horseradish-peroxidase-conjugated anti-mouse (1:15,000; #1705047, BioRad) or anti-rabbit (1:15,000; #1705046, BioRad) for 1 hour at room temperature. Membranes were developed using ECL Clarity reagent (BioRad) and imaged using ChemiDoc MP Imaging System and Image Lab software (BioRad). Band intensities were quantified using ImageJ software as described previously^15^.

For TIMP2 detection in ISF samples, 4 consecutive baseline eISF samples from microdialysis were pooled and concentrated ~6 fold using Amicon Ultra Centrifugal Filters (3 kDa Ultracel, 0.5 mL, Millipore). Concentrated samples were then processed for western blotting as described above. Estimation of exchangeable interstitial fluid (eISF) TIMP2 protein levels were determined by extrapolation from a standard curve of recombinant mouse TIMP2 (R&D/Biotechne) ranging from 0.31 ng to 20 ng run alongside ISF samples in SDS-PAGE/Western that had been concentrated ~9.2-fold.

**
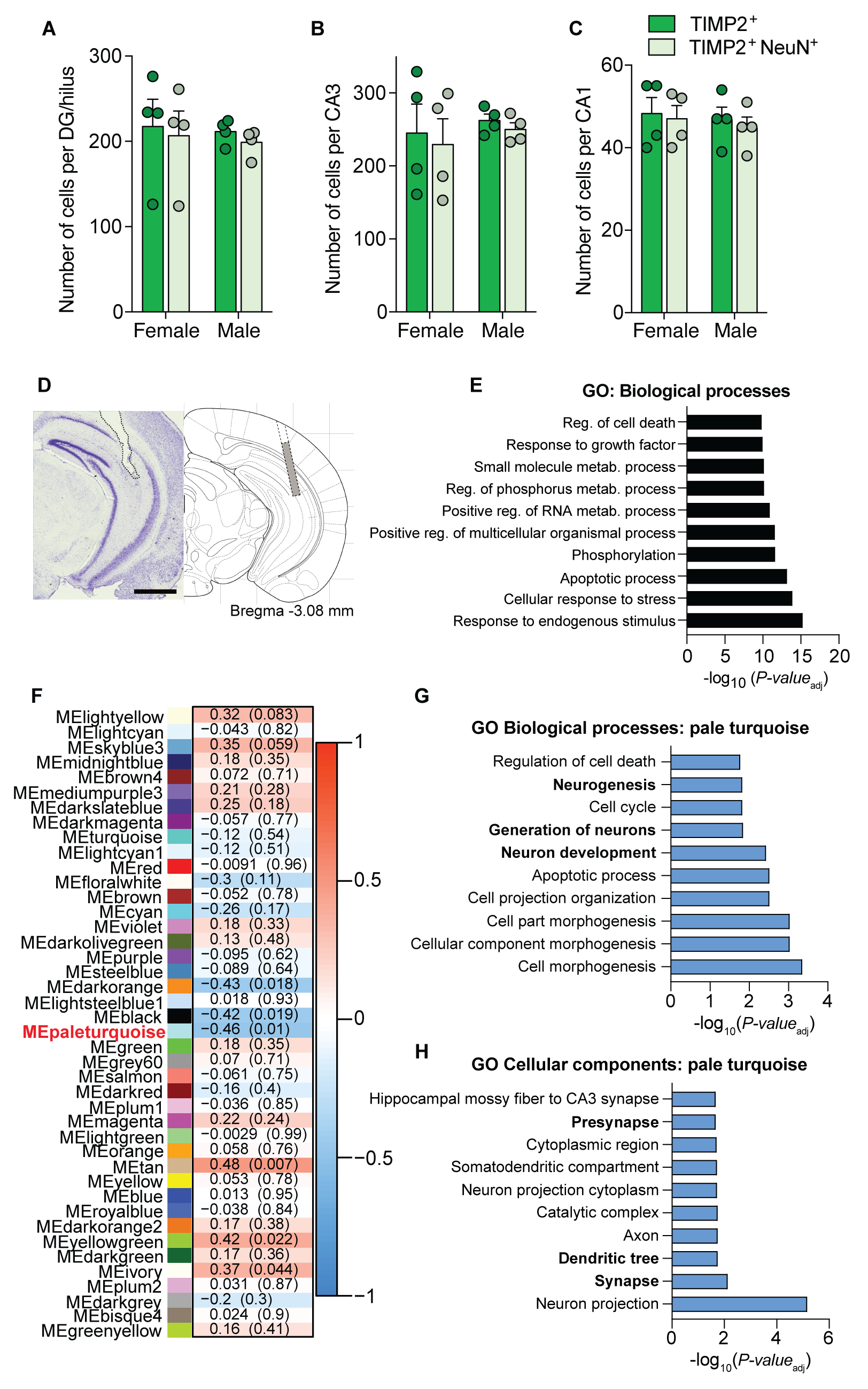
Supplementary Figure 1**

**Supplementary Figure 1. TIMP2 protein is found in hippocampal neurons in male and female mice, and its loss affects hippocampal transcriptome.**

**(A)** Quantification of the total number of TIMP2^+^ and TIMP2^+^ NeuN^+^ cells at the hilus/DG, **(B)** CA3 and **(C)** CA1 subregions of the hippocampus from WT males and females at 2 months of age (N = 4 mice per sex). **(D)** Cresyl violet-stained section depicting the microdialysis probe tract through brain surface extending into hippocampus, with microdialysis probe position seen at Bregma -3.08mm based on Paxinos and Watson atlas. Scale bar, 500 μm. Data are represented as mean ± SEM. **(E)** Top 10 pathways, ranked by significance, for upregulated DEGs in hippocampi of TIMP2 KO mice relative to WT mice (N = 13-17 mice per group). **(F)** Module-trait relationships plot for all module assignments (labeled by color in leftmost column), with corresponding module membership values and respective module significance (denoted in parentheses; (alpha=0.05)) based on WGCNA from genes measured by RNA-seq in TIMP2 KO and WT hippocampi (Rightmost color bar refers to modules with upregulated (red) and downregulated (blue) genes. **(G)** Top 10 significant GO Biological Processes and **(H)** Cellular Component pathways following GSEA on “pale turquoise” module from module trait analysis.

**Supplementary Figure 2**


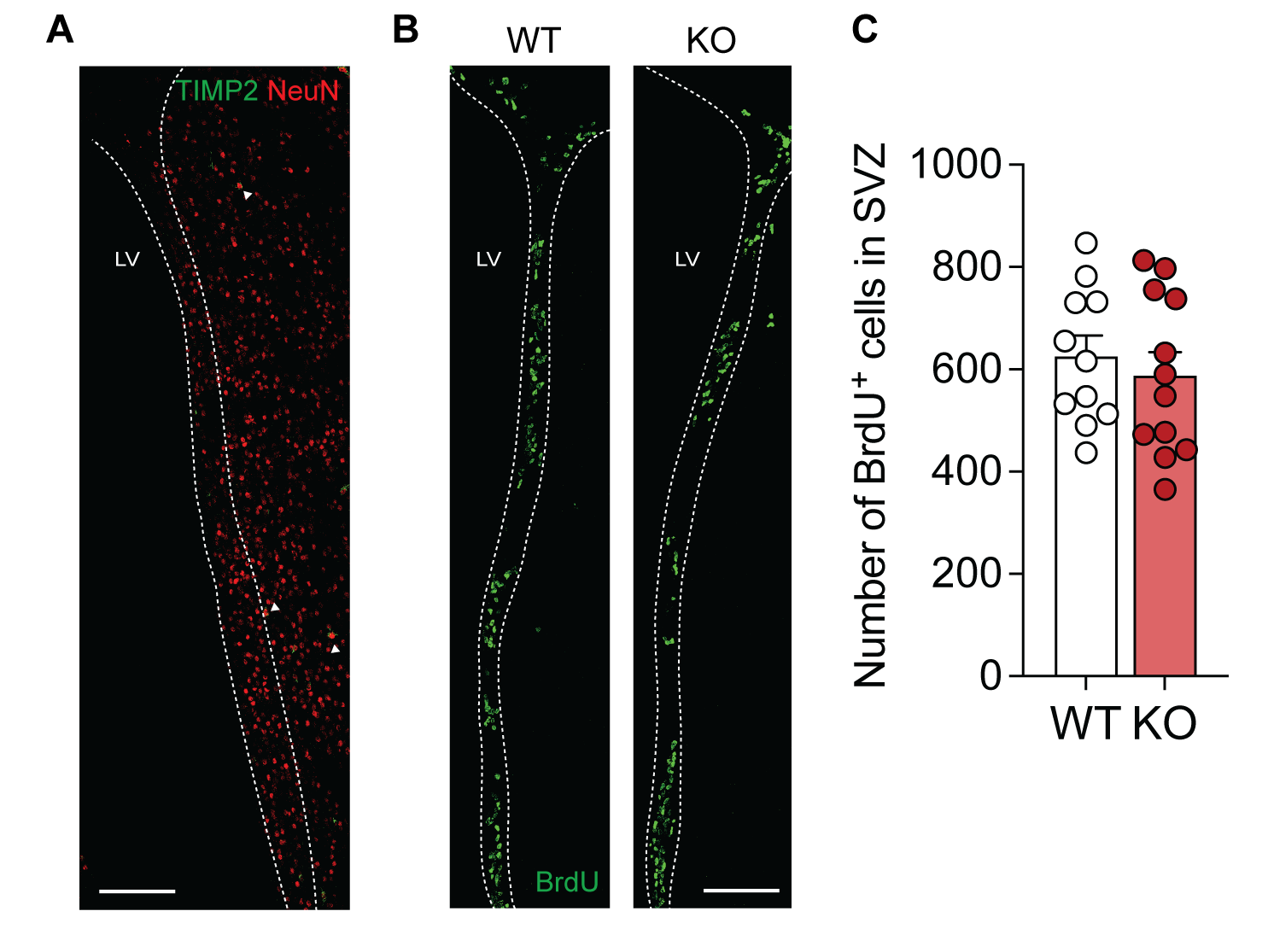


**Supplementary Figure 2: (A)** Representative confocal image of 2-month-old WT SVZ (N = 5 mice) showing few TIMP2^+^ cells; scale bar, 100 μm. **(B)** Representative confocal images of BrdU staining in the subventricular zone (SVZ) of WT and TIMP2 KO (2-3 months of age, N = 11-12 mice per group; scale bar, 100 μm), with corresponding **(C)** quantification of number of BrdU^+^ cells in SVZ of WT and TIMP2 KO mice. Data are represented as mean ± SEM. Data points represent individual mice. SVZ, subventricular zone; LV, lateral ventricle.


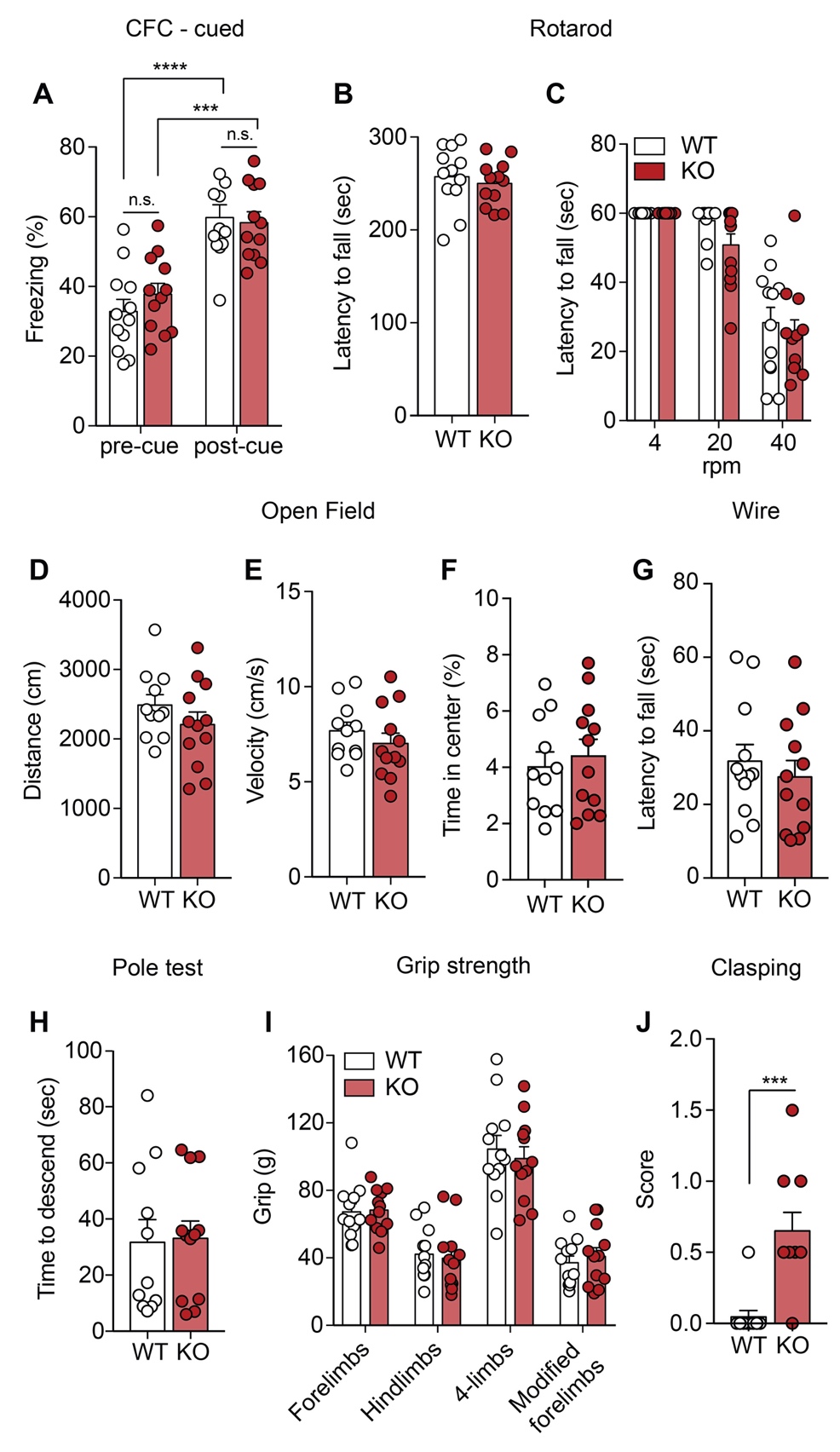
**Supplementary Figure 3**

**Supplementary Figure 3. Loss of TIMP2 does not affect amygdala-associated behavior or overall motor function.**

**(A)** Percentage of freezing detected in the cued task of the fear-conditioning assay in WT and TIMP2 KO mice (2-3 months of age, N = 12 mice per group). **(B)** Latency of TIMP2 KO and WT mice to fall in the rotarod in the fixed and **(C)** acceleration protocol at 4, 20 and 40 rpm (N = 12 mice per group). **(D)** Total distance traveled by TIMP2 KO and WT mice in the open field, as well as **(E)** velocity, and **(F)** percentage of time spent in the center of the arena (N = 11-12 mice per group). **(G)** Latency of TIMP2 KO and WT mice to fall in the wire test (N = 12 mice per group). **(H)** Time for TIMP2 KO and WT mice to descend the pole in the pole test (N = 11-12 mice per group). **(I)** Grip strength of the fore-, hind- and four limbs in TIMP2 KO and WT mice (N = 12 mice per group). **(J)** Hindlimb extension by clasping score in TIMP2 KO and WT mice (N = 10-11 mice per group). Data are represented as mean ± SEM. Student’s *t*-test for two-group comparisons. ****P*<0.001, *****P*<0.0001, n.s., not significant.

**
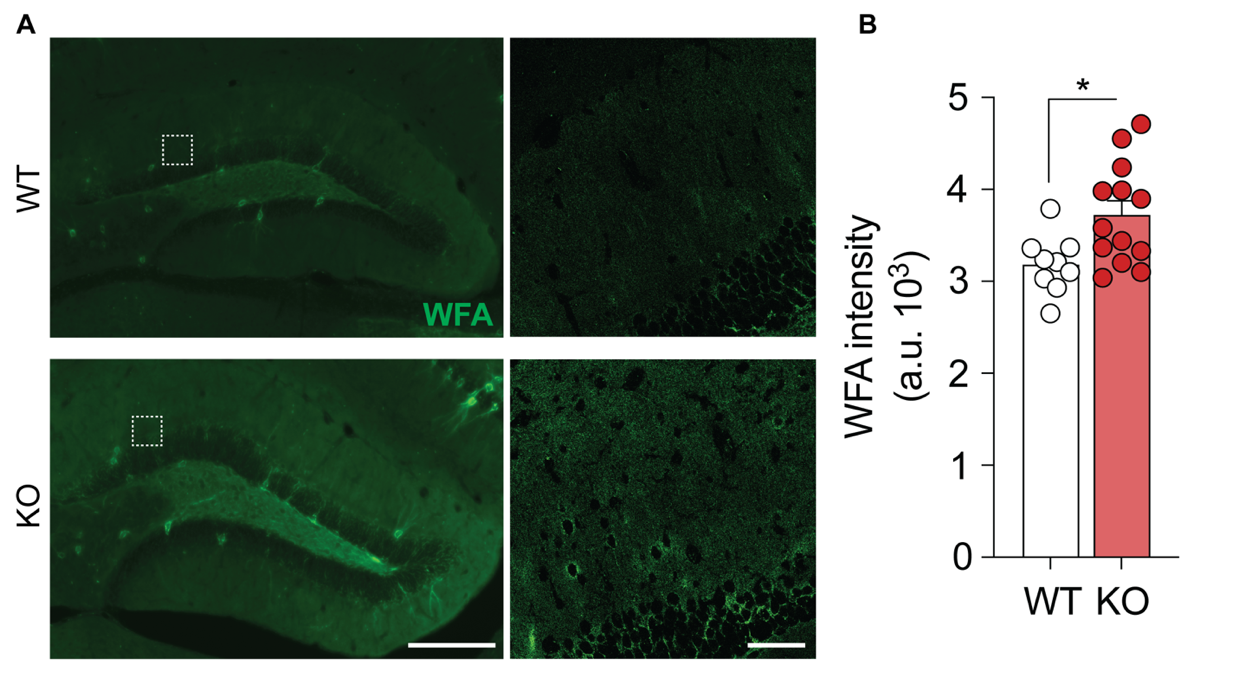
Supplementary Figure 4**

**Supplementary Figure 4: TIMP2 deletion leads to alterations in perineuronal nets within the hippocampus.** (**A**) Representative confocal microscopy images of *Wisteria floribunda* agglutinin (WFA) staining in the DG of WT and TIMP2 KO mice (2-3 months of age, N = 9-13 mice per group; white box depicts sample ROI; scale bar for left image column, 500 μm; scale bar for right image column, 50 μm). (**B**) Quantification of WFA intensity in the DG of WT and TIMP2 KO mice (a.u. = arbitrary units). Data are represented as mean ± SEM, and data points represent individual mice. Student’s t-test, **P*<0.05.

**Supplementary Figure 5**


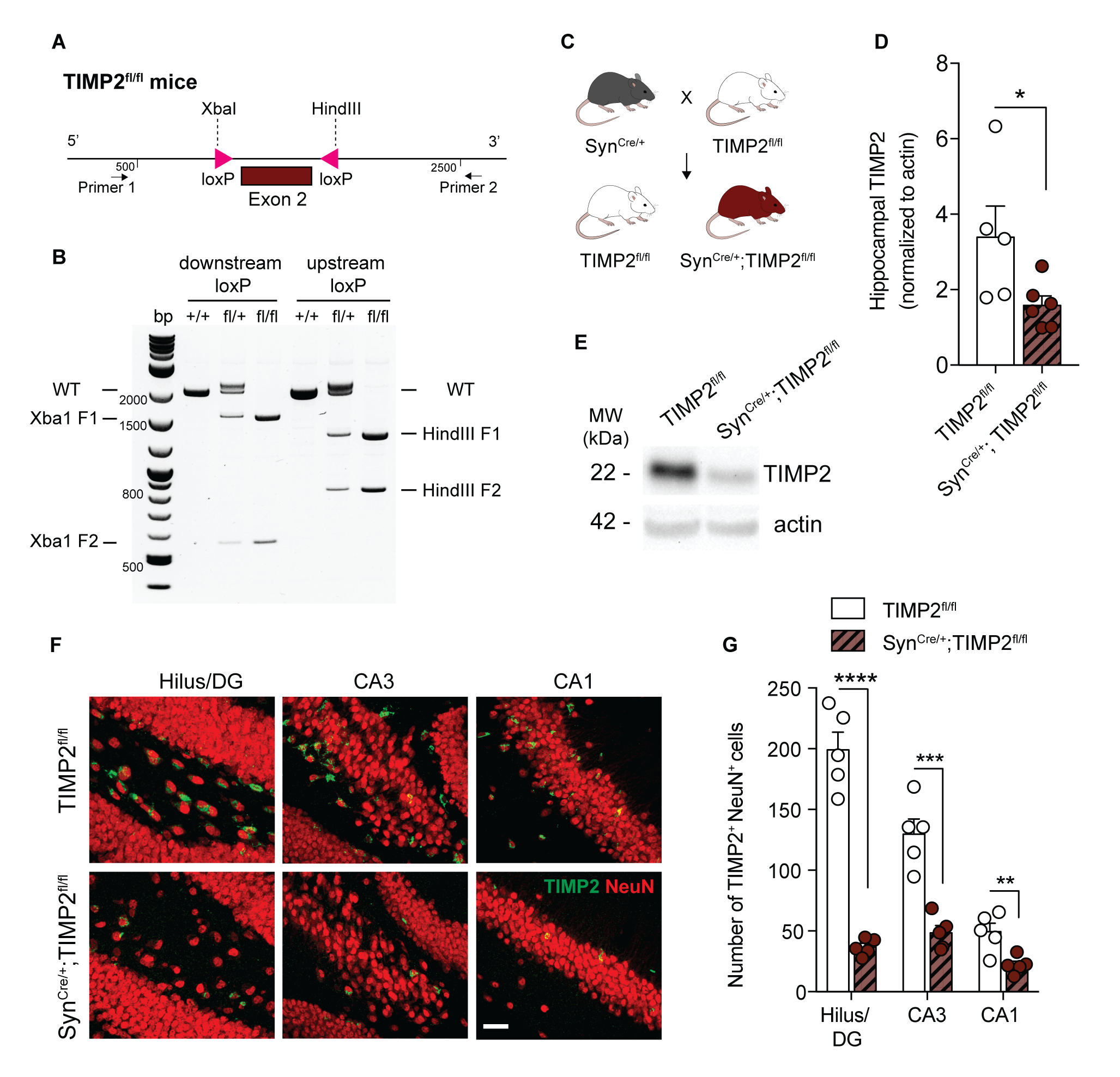


**Supplementary Figure 5. Creation of a conditional model to target neuronal pools of TIMP2.**

**(A)** Schematic illustration of the targeting strategy of to insert loxP sites flanking exon 2 to generate a model for conditional deletion of *TIMP2*. **(B)** Representative genotyping results revealing the PCR products for the mutant LoxP and wild-type alleles. Wild-type (+/+), heterozygous (fl/+), and homozygous (fl/fl) mice were identified according to this strategy. **(C)** Schematic diagram of cross-breeding strategy to establish neuron-specific TIMP2 deletion: male TIMP2^fl/fl^ mice were mated with Syn^Cre/+^ females to obtain Syn^Cre/+^; TIMP2^fl/fl^ and their respective TIMP2^fl/fl^ littermate controls. **(D-E)** Representative TIMP2 immunoblot and corresponding quantification of TIMP2 protein levels from hippocampal lysate of TIMP2^fl/fl^ and Syn^Cre/+^; TIMP2^fl/fl^ (2-3 months of age, N = 5-6 mice per group). **(F)** High-magnification view of hilus/DG, CA3, and CA1 sub-regions of TIMP2^+^ cells co-expressing NeuN in TIMP2^fl/fl^ and Syn^Cre/+^; TIMP2^fl/fl^ mice (2-3 months of age, N = 5 mice per group, scale bar, 20 μm) with corresponding **(G)** quantification of the total number of TIMP2^+^ cells with NeuN^+^ nuclei across hippocampal subregions. Data are represented as mean ± SEM. Student’s t-test for two-group comparisons. **P*<0.05, ***P*<0.01, ****P*<0.001, *****P*<0.0001. Data points represent individual mice.

**Supplementary Figure 6**


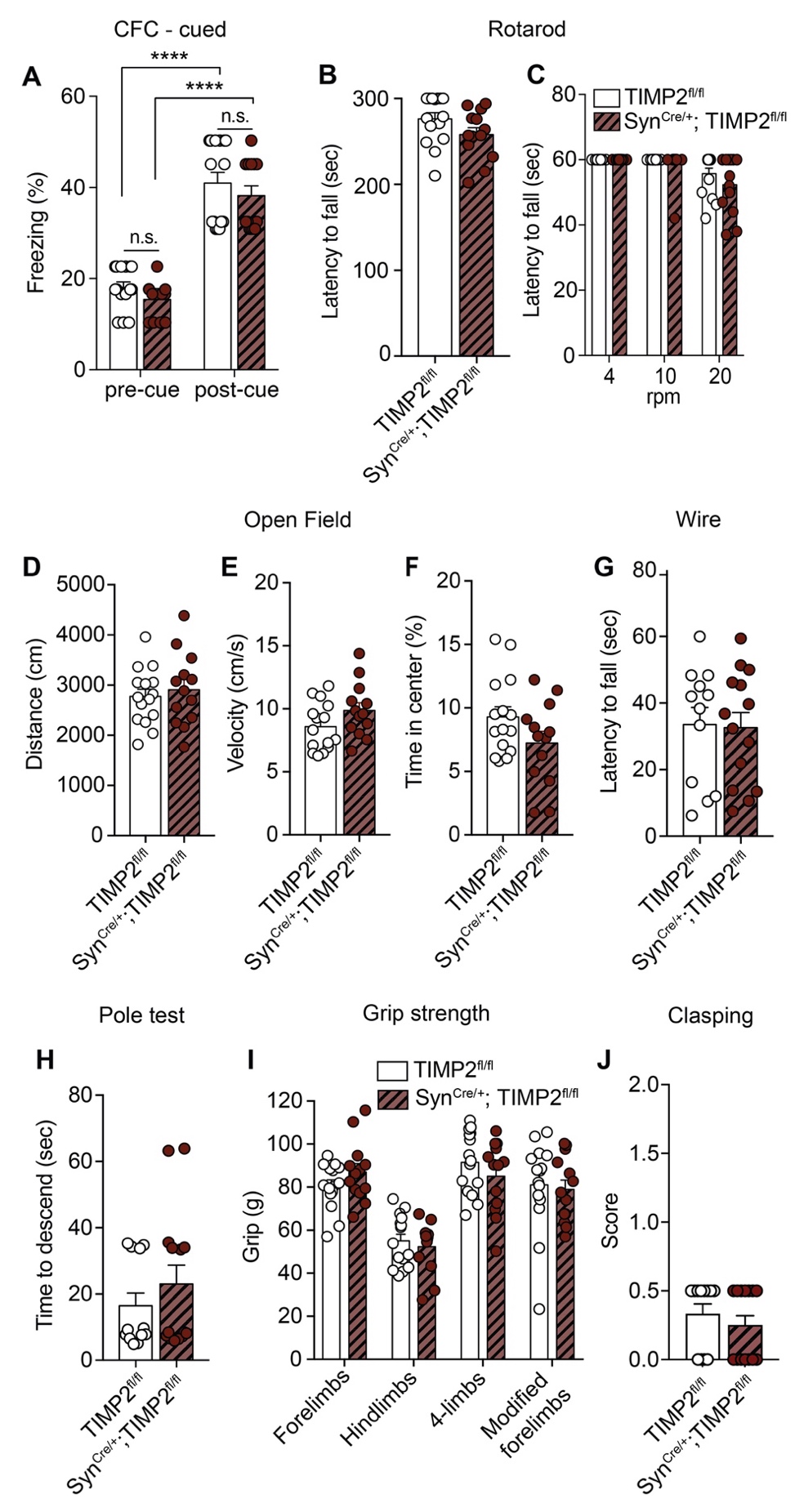


**Supplementary Figure 6. Neuronal TIMP2 deletion does not impair amygdala-associated behavior or overall motor function.**

**(A)** Percentage of freezing detected in the cued task of the fear-conditioning assay in TIMP2^fl/fl^ and Syn^Cre/+^; TIMP2^fl/fl^ mice (2-3 months of age, N = 13-15 mice per group). **(B)** Latency of TIMP2^fl/fl^ and Syn^Cre/+^; TIMP2^fl/fl^ mice to fall in the rotarod in the fixed and **(C)** acceleration protocol at 4, 10 and 20 rpm (N = 13-15 mice per group). **(D)** Total distance traveled by TIMP2^fl/fl^ and Syn^Cre/+^; TIMP2^fl/fl^ mice in the open field, as well as **(E)** velocity, and **(F)** percentage of time spent in the center of the arena (N = 13-15 mice per group). **(G)** Latency of TIMP2^fl/fl^ and Syn^Cre/+^; TIMP2^fl/fl^ mice to fall in the wire test (N = 12-14 mice per group). **(H)** Time for TIMP2^fl/fl^ and Syn^Cre/+^; TIMP2^fl/fl^ mice to descend the pole in the pole test (N = 12-14 mice per group). **(I)** Grip strength of the fore-, hind- and four limbs in TIMP2^fl/fl^ and Syn^Cre/+^; TIMP2^fl/fl^ mice (N = 13-15 mice per group). **(J)** Hindlimb extension by clasping score in TIMP2^fl/fl^ and Syn^Cre/+^; TIMP2^fl/fl^ mice (N = 12-14 mice per group). Data are represented as mean ± SEM. Student’s *t*-test for two-group comparisons. n.s., not significant.

**Supplementary References**

1. Gu B, Posfai E, Rossant J. Efficient generation of targeted large insertions by microinjection into two-cell-stage mouse embryos. *Nat Biotechnol* 2018; **36**(7)**:** 632-637.

2. Zhu Y, Romero MI, Ghosh P, Ye Z, Charnay P, Rushing EJ *et al.* Ablation of NF1 function in neurons induces abnormal development of cerebral cortex and reactive gliosis in the brain. *Genes Dev* 2001; **15**(7)**:** 859-876.

3. Ulrich JD, Burchett JM, Restivo JL, Schuler DR, Verghese PB, Mahan TE *et al.* In vivo measurement of apolipoprotein E from the brain interstitial fluid using microdialysis. *Mol Neurodegener* 2013; **8:** 13.

4. Smith AC, Kupchik YM, Scofield MD, Gipson CD, Wiggins A, Thomas CA *et al.* Synaptic plasticity mediating cocaine relapse requires matrix metalloproteinases. *Nat Neurosci* 2014; **17**(12)**:** 1655-1657.

5. Jiang M, Tu HT, Zhang K, Zhang W, Yu WP, Xu J *et al.* Impaired neurogenesis in the hippocampus of an adult VPS35 mutant mouse model of Parkinson's disease through interaction with APP. *Neurobiol Dis* 2021; **153:** 105313.

6. Castellano JM, Mosher KI, Abbey RJ, McBride AA, James ML, Berdnik D *et al.* Human umbilical cord plasma proteins revitalize hippocampal function in aged mice. *Nature* 2017; **544**(7651)**:** 488-492.

7. Nguyen PT, Dorman LC, Pan S, Vainchtein ID, Han RT, Nakao-Inoue H *et al.* Microglial Remodeling of the Extracellular Matrix Promotes Synapse Plasticity. *Cell* 2020; **182**(2)**:** 388-403 e315.

8. Ippolito DM, Eroglu C. Quantifying synapses: an immunocytochemistry-based assay to quantify synapse number. *J Vis Exp* 2010; (45).

9. Jacot-Descombes S, Keshav NU, Dickstein DL, Wicinski B, Janssen WGM, Hiester LL *et al.* Altered synaptic ultrastructure in the prefrontal cortex of Shank3-deficient rats. *Mol Autism* 2020; **11**(1)**:** 89.

10. De Waele J, Reekmans K, Daans J, Goossens H, Berneman Z, Ponsaerts P. 3D culture of murine neural stem cells on decellularized mouse brain sections. *Biomaterials* 2015; **41:** 122-131.

11. Tajerian M, Hung V, Nguyen H, Lee G, Joubert LM, Malkovskiy AV *et al.* The hippocampal extracellular matrix regulates pain and memory after injury. *Mol Psychiatry* 2018; **23**(12)**:** 2302-2313.

12. Mateus-Pinheiro A, Alves ND, Patricio P, Machado-Santos AR, Loureiro-Campos E, Silva JM *et al.* AP2gamma controls adult hippocampal neurogenesis and modulates cognitive, but not anxiety or depressive-like behavior. *Mol Psychiatry* 2017; **22**(12)**:** 1725-1734.

13. Zhu JW, Li YF, Wang ZT, Jia WQ, Xu RX. Toll-Like Receptor 4 Deficiency Impairs Motor Coordination. *Front Neurosci* 2016; **10:** 33.

14. Takeshita H, Yamamoto K, Nozato S, Inagaki T, Tsuchimochi H, Shirai M *et al.* Modified forelimb grip strength test detects aging-associated physiological decline in skeletal muscle function in male mice. *Sci Rep* 2017; **7:** 42323.

15. Castellano JM, Kim J, Stewart FR, Jiang H, DeMattos RB, Patterson BW *et al.* Human apoE isoforms differentially regulate brain amyloid-beta peptide clearance. *Sci Transl Med* 2011; **3**(89)**:** 89ra57.
